# Supplementary material for: MAPT Genetic Variation and Neuronal Maturity Alter Isoform Expression Affecting Axonal Transport in iPSC-Derived Dopamine Neurons
Source: Stem Cell Reports. 2017 Jul 6;9(2):587–99. doi: 10.1016/j.stemcr.2017.06.005 (PMC5549835; doi:10.1016/j.stemcr.2017.06.005)
Supplement: Document S1. Supplemental Experimental Procedures, Figures S1–S6, and Table S1 [file mmc1.pdf]

Stem Cell Reports, Volume *9*

Supplemental Information

MAPT Genetic Variation and Neuronal Maturity Alter Isoform Expression Affecting Axonal Transport in iPSC-Derived Dopamine Neurons

Joel E. Beevers, Mang Ching Lai, Emma Collins, Heather D.E. Booth, Federico Zambon, Laura Parkkinen, Jane Vowles, Sally A. Cowley, Richard Wade-Martins, and Tara M. Caffrey

Supplemental Information

Figure S1

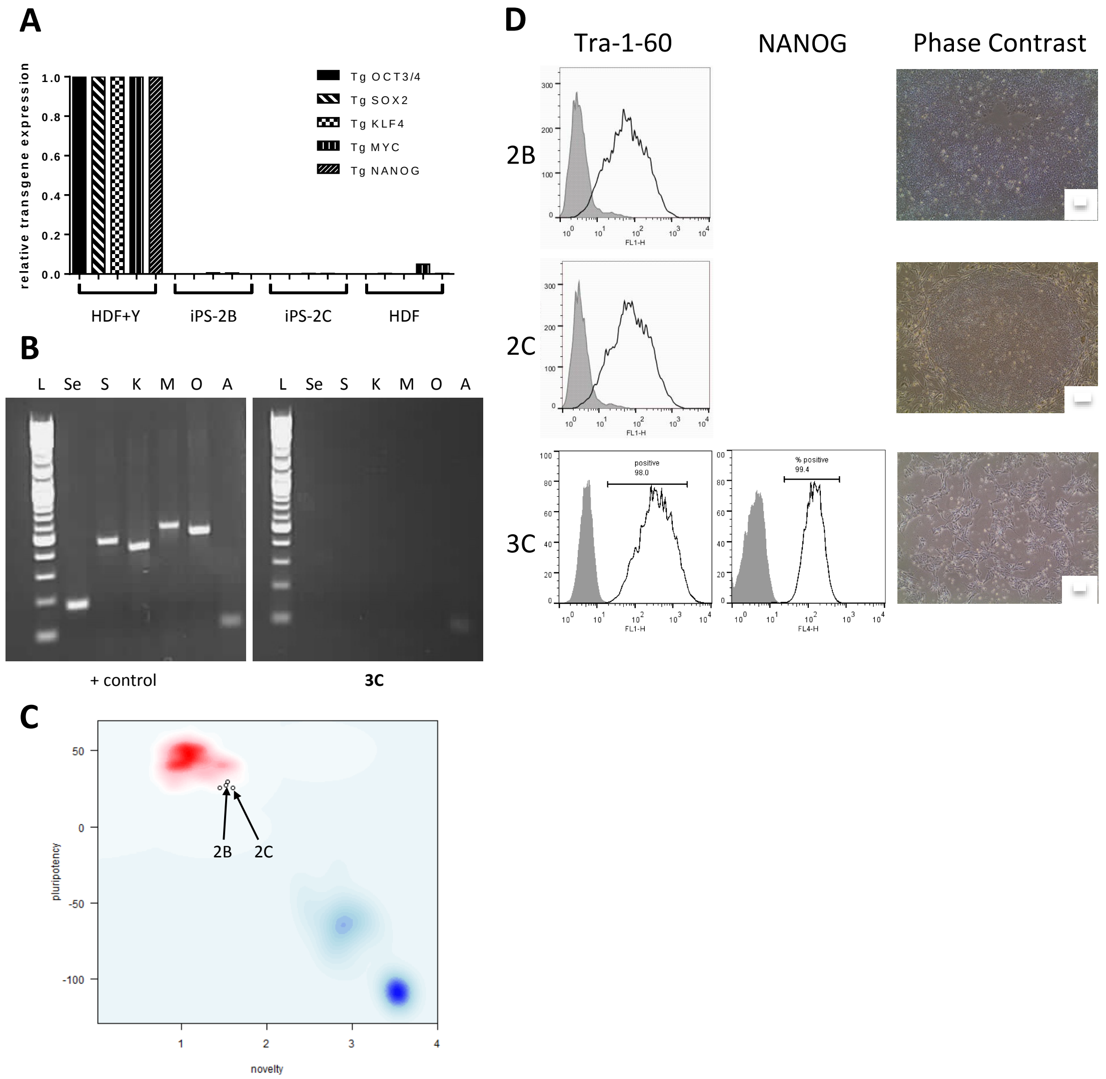

Figure S1: Characterisation of previously unpublished iPSC clones 2B, 2C and 3C, related to Experimental Procedures and Figure 1.

(A) Retroviral transgene silencing in iPSC lines 2B and 2C by qRT-PCR for each transgene, normalised to actin endogenous control, relative to expression of transgenes from fibroblasts 5 days post-infection with the Yamanaka reprogramming retroviruses (HDF+Y), with uninfected fibroblasts (HDF) as a negative control.

(B) CytoTune Sendai virus clearance shows the correct size band for β-actin, and no bands corresponding to the reprogramming virus RT-PCR product sizes for iPSC line 3C; L, Log2 ladder; Se, Sendai backbone 181 bp; S, Sox2 451 bp; K, Klf4 410 bp; M, c-myc 532 bp; O, Oct-4 483 bp; A, β-actin control 92 bp; + control, fibroblasts infected with CytoTune 5 days previously.

(C) PluriTest analysis of Illumina HT12v4 transcriptome array data shows the tested iPSC lines cluster in the red cloud representing pluripotent cell lines and not with differentiated cells (blue clouds).

(D) iPSC lines express expected pluripotency proteins as shown by FACs for Tra-1-60 and Nanog (grown feeder-free); open black plot represents antibody, filled grey plot is isotype control; Right-hand panel shows the expected iPSC colony morphology, with high nucleus to cytoplasm ratio by phase-contrast microscopy; 2B is photographed on matrigel, 2C on feeders, 3C photographed one day after thawing onto matrigel, so not yet clustered tightly together. Scale bar = 100 µm.

Figure S2

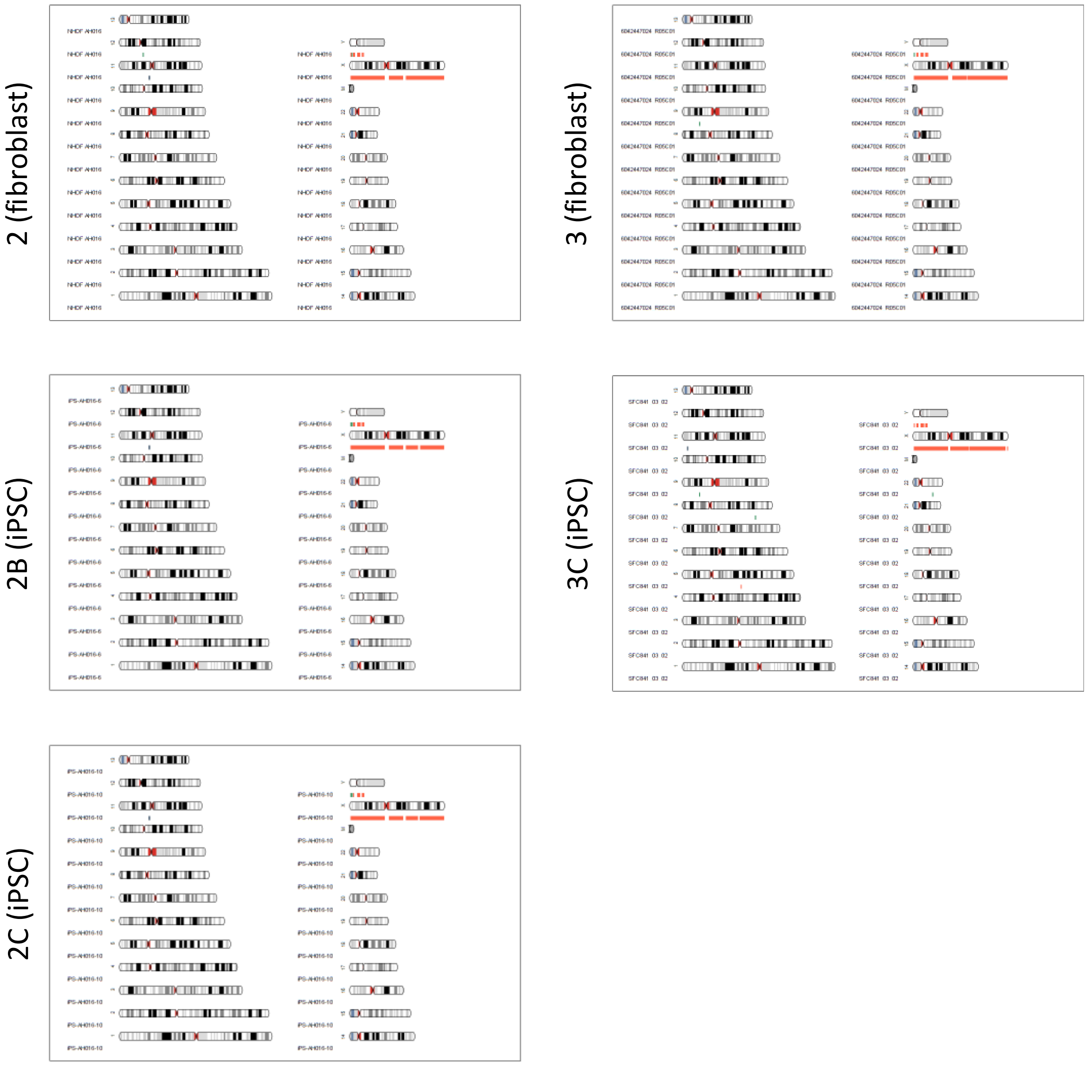

Figure S2: Further characterisation of previously unpublished iPSC clones 2B, 2C and 3C, related to Experimental Procedures and Figure 1.
Genome integrity was assessed by Illumina Human CytoSNP-12v2.1 or OmniExpress24 SNP array and karyograms produced using KaryoStudio software (Illumina); autosome amplifications (green), deletions (orange) and LOH regions (grey) are shown alongside the relevant chromosome; single-copy sex chromosomes are annotated orange, X chromosomes for females are grey.

Figure S3

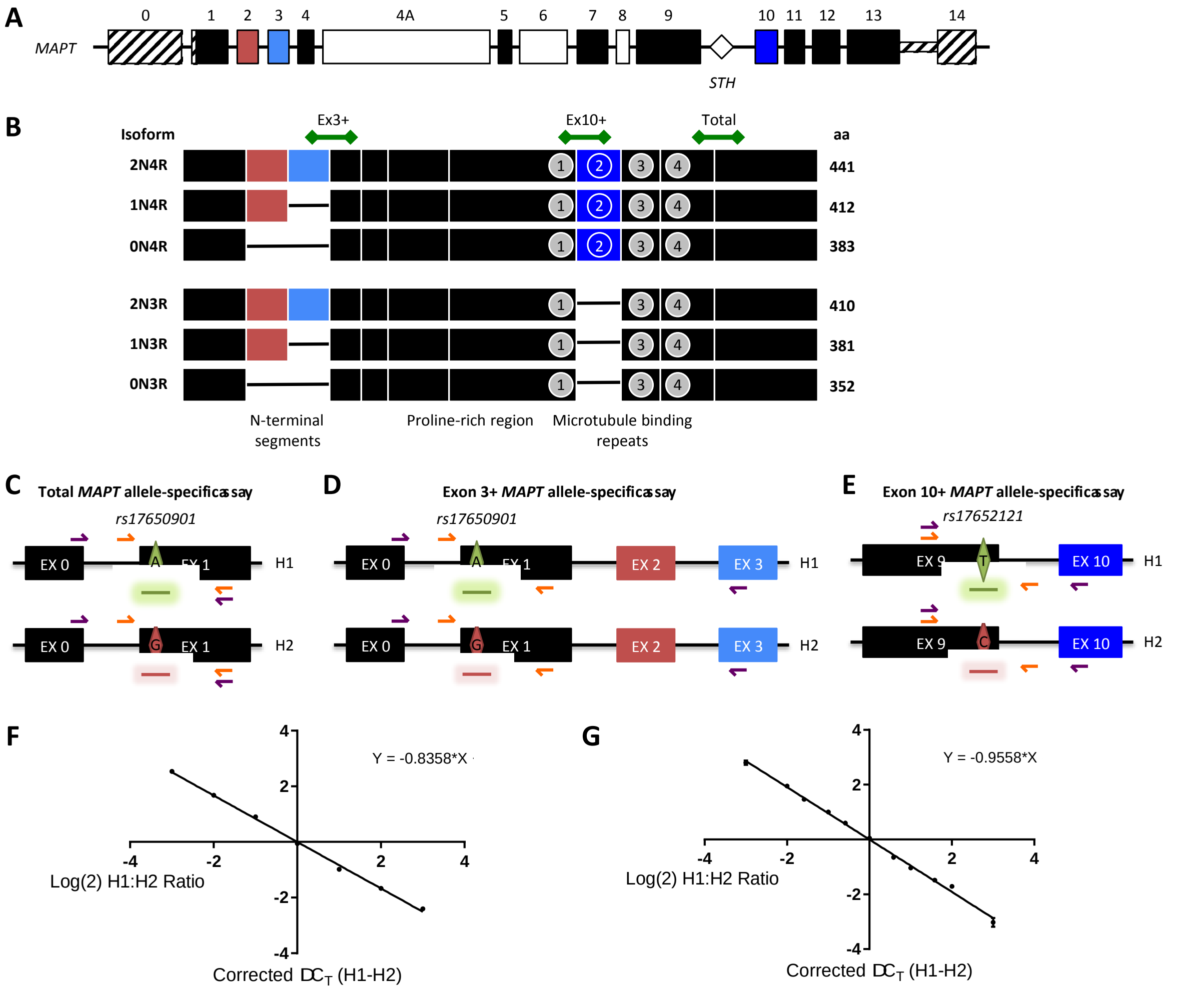

Figure S3: Schematics of the human *MAPT* locus and quantitative RT-PCR assays, related to Figures 2 and 4.

(A) The human *MAPT* locus on chromosome 17q21.31 contains 16 exons; exons 2, 3 and 10 (coloured) are subject to alternative splicing, exons 9-12 encode microtubule-binding domains with a high degree of sequence similarity and exons 4A, 6 and 8 (white) are not expressed in the adult central nervous system.

(B) Adult human brain expresses six major isoforms of tau protein through the alternative splicing of exons 2, 3 and 10. The locations of the standard (not allele-specific) TaqMan-based quantitative RT-PCR expression assays used in this study are shown in green.

(C-E) Schematics of the allele-specific TaqMan-based expression assays exploiting haplotype-tagging SNPs to distinguish the allelic origin of transcripts, with the H1 or H2 allele shown respectively as a green or red diamond. TaqMan probes bearing FAM (green) or VIC (red) are shown as coloured lines below the SNP. Arrows denote primers to amplify from genomic DNA (orange) or cDNA (purple). (C) Allele-specific assay for total *MAPT* transcripts using SNP rs17650901 (SNP1) in exon 1. (D) Allele-specific assay for exon 3+ *MAPT* transcripts also using SNP rs17650901 (SNP1) in exon 1. (E) Allele-specific assay for exon 10+ *MAPT* transcripts using SNP rs17652121 in exon 9 (SNP9ii).

(F-G) Standard curves for (F) the allele-specific expression assay using SNP1 (rs17650901) and (G) the allele-specific expression assay using SNP9ii (rs17652121). Standard curves were generated using *MAPT* H1 PAC and *MAPT* H2 PAC in ratios from 8:1 to 1:8. The values of ΔC_T (H1-H2) were plotted against the log(2) H1:H2 ratio, then corrected to remove the value of the y-intercept (to bring the graph through the origin).

Figure S4

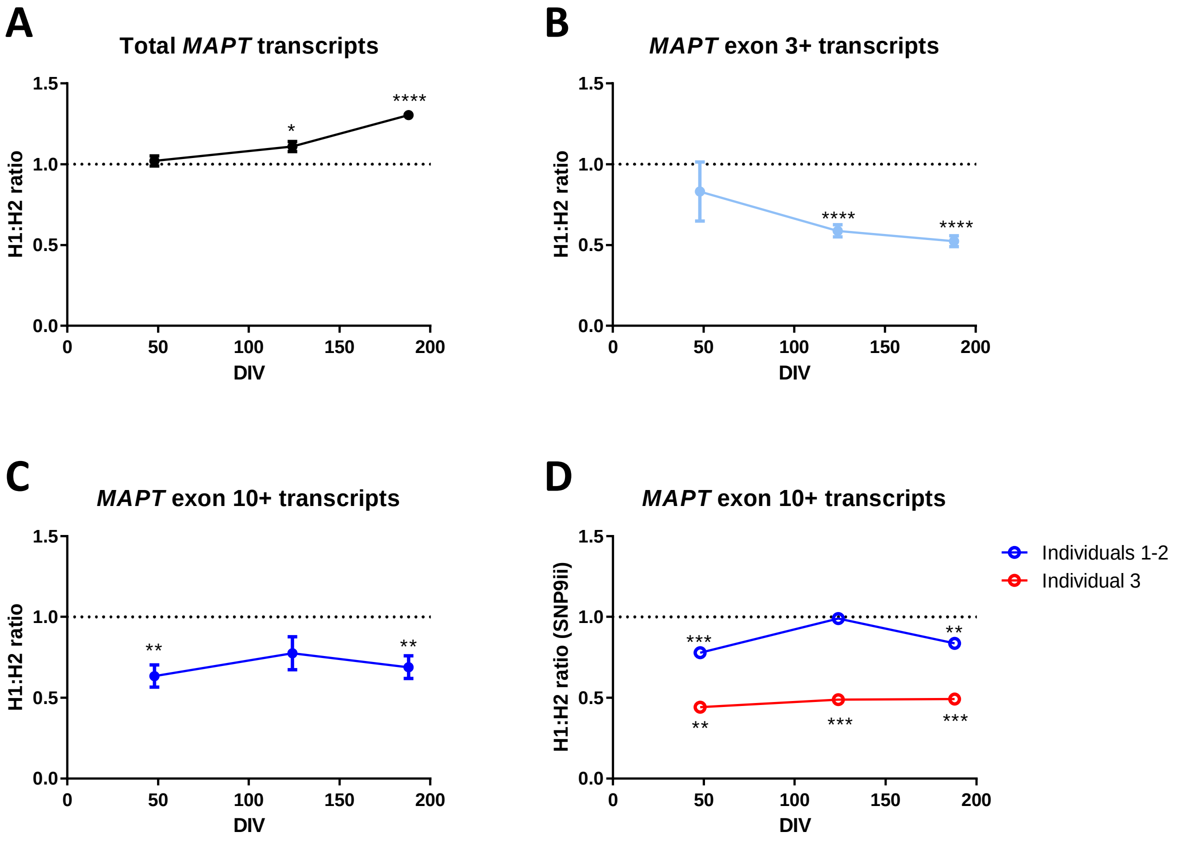

Figure S4: Allele-specific *MAPT* expression assays on dopaminergic neuronal cultures over 24-week maturation, related to Figure 4.

(A-C) Data from allele-specific TaqMan-based quantitative RT-PCR expression assays on samples from three points of a time course of maturation of dopaminergic neuronal cultures: DIV48, DIV124 and DIV188. Each graph shows mean ±SEM, n=7 clones (all clones except clone 1A). Asterisks denote significant statistical difference from hypothetical mean of 1 in a one-sample t-test: (A) DIV48, mean=1.020 ± 0.031, n.s.; DIV124, mean=1.109 ± 0.031, $p=0.0132$, $t=3.478$, df=6; DIV188, mean=1.305 ± 0.024, $p<0.0001$, $t=12.58$, df=6; (B) DIV48, mean=0.832 ± 0.183, n.s.; DIV124, mean=0.588 ± 0.038, $p<0.0001$, $t=10.97$, df=6; DIV188, mean=0.524 ± 0.034, $p<0.0001$, $t=13.95$, df=6; (C) DIV48, mean=0.635 ± 0.069, $p=0.0019$, $t=5.280$, df=6; DIV124, mean=0.776 ± 0.103, n.s.; DIV188, mean=0.689 ± 0.070, $p=0.0044$, $t=4.437$, df=6.

(D) Subdivision of graph C to separate out individual 3, which consistently showed a distinct phenotype relative to exon 10. Individuals 1-2, n=4 clones; individual 3, n=3 clones. Asterisks denote significant statistical difference from hypothetical mean of 1 in a one-sample t-test: individuals 1-2 DIV48, mean=0.780 ± 0.013, $p=0.0005$, $t=16.50$, df=3; individuals 1-2 DIV124, mean=0.991 ± 0.030, n.s.; individuals 1-2 DIV188, mean=0.837 ± 0.014, $p=0.0013$, $t=11.74$, df=3; individual 3 DIV48, mean=0.441 ± 0.019, $p=0.0011$, $t=29.85$, df=2; individual 3 DIV124, mean=0.488 ± 0.006, $p=0.0002$, $t=81.41$, df=2; individual 3 DIV188, mean=0.492 ± 0.006, $p=0.0002$, $t=81.39$, df=2.

Figure S5

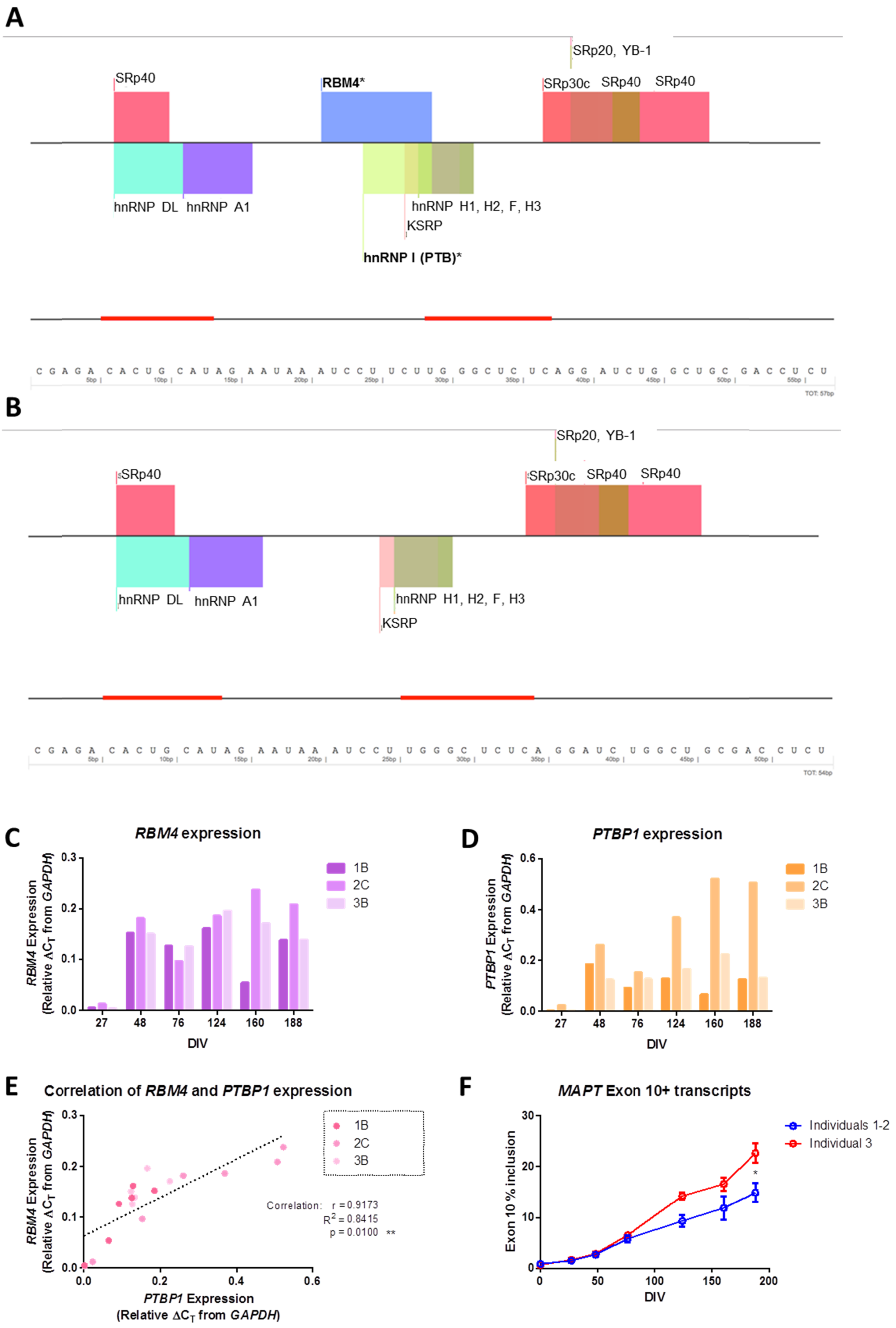

A
SRp20, YB-1
SRp40
RBM4*
SRp30c
SRp40
SRp40
hnRNP DL
hnRNP A1
hnRNP H1, H2, F, H3
KSRP
hnRNP I (PTB)*
CGAGA CACUG CAUAG AAUAA AUCCU UCUUG GGCUC UCAGG AUCUG GCUGC GACCU CU
TOT: 57bp
B
SRp20, YB-1
SRp40
SRp30c
SRp40
SRp40
hnRNP DL
hnRNP A1
hnRNP H1, H2, F, H3
KSRP
CGAGA CACUG CAUAG AAUAA AUCCU UGGGC UCUCA GGAUC UGGCU GCGAC CUCU
TOT: 54bp
C
RBM4 expression
RBM4 Expression (Relative ΔC_T from GAPDH)
1B
2C
3B
DIV
D
PTBP1 expression
PTBP1 Expression (Relative ΔC_T from GAPDH)
1B
2C
3B
DIV
E
Correlation of RBM4 and PTBP1 expression
RBM4 Expression (Relative ΔC_T from GAPDH)
PTBP1 Expression (Relative ΔC_T from GAPDH)
Correlation: r = 0.9173
R² = 0.8415
p = 0.0100 **
F
MAPT Exon 10+ transcripts
Exon 10 % inclusion
DIV
Individuals 1-2
Individual 3

Figure S5: Splice factor binding site and expression analysis, related to Figure 5. Screen shots of splice factor binding analysis with *Splice Aid 2* online tool (http://193.206.120.249/splicing_tissue.html, version February 2013, 71 splicing factors, 2339 RNA binding sites, accessed February 2016) (Piva *et al.* 2012) using an input of **(A)** a 57 bp portion of the consensus sequence of *MAPT* intron 10 and **(B)** the equivalent 54 bp ΔCTT sequence as in control 3. Splice factors that promote exon inclusion or exclusion are shown as blocks respectively above or below the central line. Unlike the consensus sequence, the ΔCTT variant sequence is not predicted to be bound by RBM4 (blue box above) or hnRNP I (PTB; light green box below). All known splicing factors were included in this search, rather than restricting by tissue type.

(C-F) Quantitative RT-PCR expression over the time course of maturation for dopaminergic neuronal cultures from DIV27 to DIV188. **(C-D)** qRT-PCR expression data for *RBM4* and *PTBP1* (PTB/hnRNP I). Both splice factors are significantly expressed at the RNA level from at least DIV48 onwards but show no correlation with the rising inclusion of exon 10 (see part D). No significant difference is observed for control 3. Bars represent mean of three technical values of n=1 cDNA sample, with each of the three individuals (one clone per individual) represented as a separate bar.

(E) Alternative representation of the data from graphs A-B to reveal correlation of the expression of *RBM4* and *PTBP1* transcripts across the time course; statistical results for Pearson correlation are presented on the graph.

(F) qRT-PCR expression data for *MAPT* exon 10+ transcripts, representing a subdivision of Fig. 2C to separate out individual 3 bearing the ΔCTT variant. Individuals 1-2, n=4 clones; individual 3, n=3 clones. Asterisk denotes significant statistical difference between groups in a multiple one-sample *t*-test with Sidak-Bonferroni multiple comparison correction: $mean_{1-2}=14.91 \pm 1.85$, $mean_3=22.66 \pm 1.98$, $p=0.000133$, $t=4.36$, df=31

Figure S6

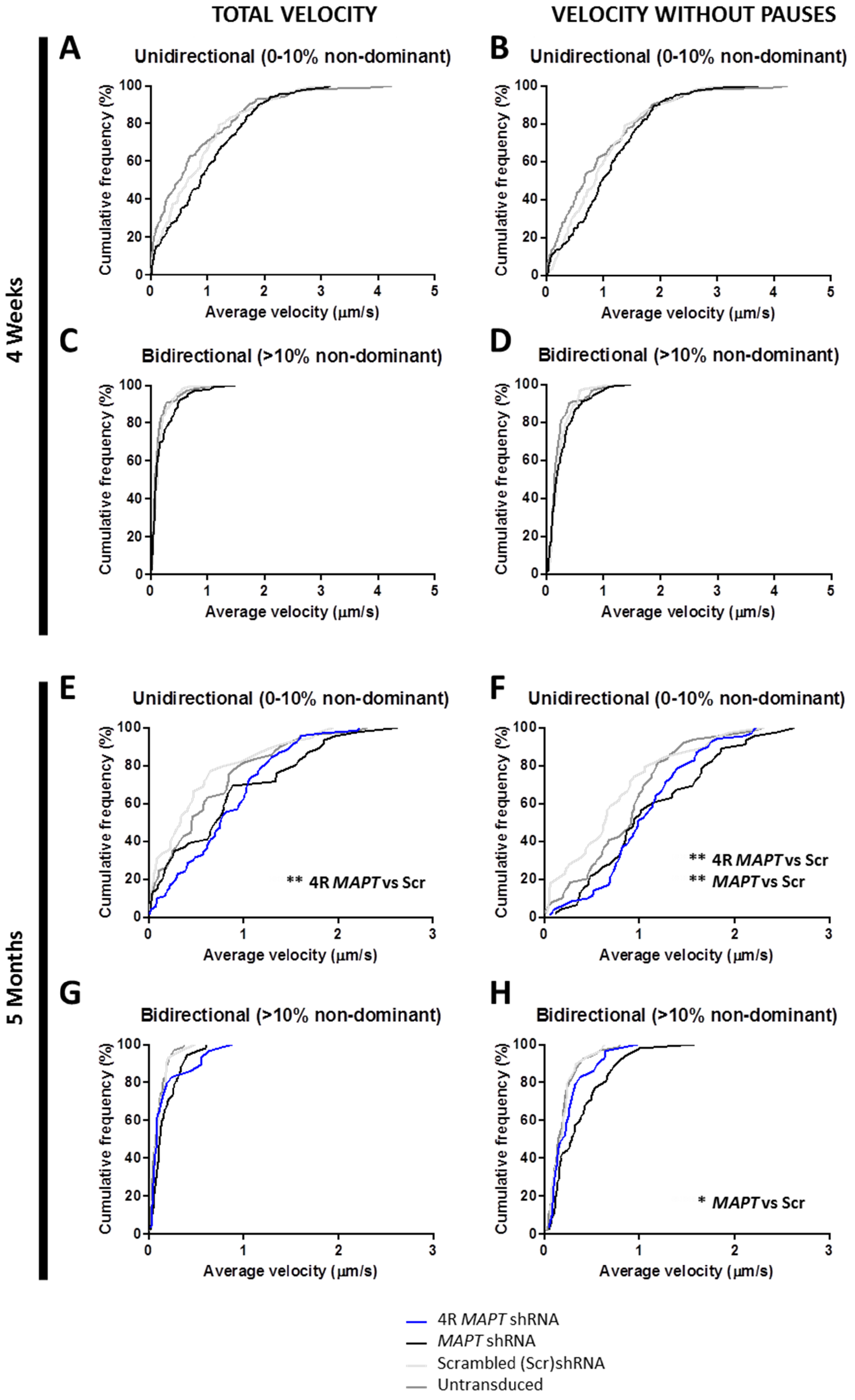
TOTAL VELOCITY
VELOCITY WITHOUT PAUSES
4 Weeks
5 Months
A
Unidirectional (0-10% non-dominant)
B
Unidirectional (0-10% non-dominant)
C
Bidirectional (>10% non-dominant)
D
Bidirectional (>10% non-dominant)
E
Unidirectional (0-10% non-dominant)
** 4R MAPT vs Scr
F
Unidirectional (0-10% non-dominant)
** 4R MAPT vs Scr
** MAPT vs Scr
G
Bidirectional (>10% non-dominant)
H
Bidirectional (>10% non-dominant)
* MAPT vs Scr
Cumulative frequency (%)
Average velocity (μm/s)
4R MAPT shRNA
MAPT shRNA
Scrambled (Scr)shRNA
Untransduced

Figure S6. Stratified mitochondrial axonal transport data for iPSC-derived dopaminergic neuronal cultures, related to Figure 6. Legend for all parts at bottom of figure. Cumulative frequency (%) graphs of average mitochondrial velocity in dopaminergic neuronal cultures after stratification by directionality.

(A-D) Stratified axonal transport data for cultures around four weeks post-transduction. (A) Total velocity measurements for unidirectional mitochondria; (B) velocity without pause periods for unidirectional mitochondria; (C) Total velocity measurements for bidirectional mitochondria; (D) velocity without pause periods for bidirectional mitochondria. Median velocities (IQR): (A) total MAPT shRNA, 0.882 (1.187) µm/s; non-targeting shRNA, 0.658 (0.908) µm/s; untransduced, 0.530 (1.139) µm/s; (B) total MAPT shRNA, 0.992 (1.011) µm/s; non-targeting shRNA, 0.869 (0.965) µm/s; untransduced, 0.671 (1.134) µm/s; (C) total MAPT shRNA, 0.096 (0.182) µm/s; non-targeting shRNA, 0.121 (0.130) µm/s; untransduced, 0.080 (0.092) µm/s; (D) total MAPT shRNA, 0.173 (0.256) µm/s; non-targeting shRNA, 0.195 (0.202) µm/s; untransduced, 0.146 (0.141) µm/s. Kruskall-Wallis test found a significant statistical difference between medians for (A) (p=0.0097, KW statistic=9.264) but Dunn's multiple comparisons post test showed no statistical difference from non-targeting shRNA control (total MAPT shRNA, adjusted p=0.2327). Kruskall-Wallis tests for (B-D) found no significant difference between medians: (B) p=0.0502, KW statistic=5.984; (C) p=0.1458, KW statistic=3.851; (D) p=0.1786, KW statistic=3.445. Number of mitochondria per condition (unidirectional/bidirectional): total MAPT shRNA, 133/102; non-targeting shRNA, 102/93; untransduced, 112/100.

(E-H) Stratified axonal transport data for cultures around five months post-transduction. (E) Total velocity measurements for unidirectional mitochondria; (F) velocity without pause periods for unidirectional mitochondria; (G) Total velocity measurements for bidirectional mitochondria; (H) velocity without pause periods for bidirectional mitochondria. Median velocities (IQR): (E) 4R *MAPT* shRNA, 0.760 (0.752) µm/s; total *MAPT* shRNA, 0.723 (1.202) µm/s; non-targeting 0.344 (0.573) µm/s; untransduced, 0.458 (0.718) µm/s; (F) 4R *MAPT* 0.990 (0.626) µm/s; total *MAPT* shRNA, 0.946 (1.056) µm/s; non-targeting 0.641 (0.827) µm/s; untransduced, 0.913 (0.660) µm/s; (G) 4R *MAPT* 0.085 (0.131) µm/s; total *MAPT* shRNA, 0.120 (0.193) µm/s; non-targeting 0.093 (0.113) µm/s; untransduced, 0.076 (0.106) µm/s; (H) 4R *MAPT* 0.214 (0.215) µm/s; total *MAPT* shRNA, 0.294 (0.389) µm/s; non-targeting 0.184 (0.154) µm/s; untransduced, 0.153 (0.145) µm/s. Kruskall-Wallis tests found significant statistical differences between medians for all graphs: (E) *p*=0.0075, KW statistic=11.97; (F) *p*=0.0030, KW statistic=13.96; (G) *p*=0.0113, KW statistic=11.09; (H) *p*=0.0022, KW statistic=14.59. Asterisks represent significant statistical difference from non-targeting shRNA control in Dunn's multiple comparisons tests: (E) 4R *MAPT* shRNA, *p*=0.0048; (F) 4R *MAPT* shRNA, *p*=0.0033; total *MAPT* shRNA, *p*=0.0078; (G) no significance in post test; (H) total *MAPT* shRNA, *p*=0.0327. Number of mitochondria per condition (unidirectional/bidirectional): 4R *MAPT* shRNA, 70/29; total *MAPT*, 52/46; non-targeting shRNA, 39/29; untransduced, 49/66.

.

Table S1: iPSC clones used in this study, Related to Figure 1.

**OPDC (StemBANCC) codes**	**Age at biopsy**	**Sex**	***MAPT* genotype**	**Clones used**	**Paper ID**	**Reprogramming Method**	**Previous Characterisation**
NHDF	44	F	H1/H2	NHDF-1<br><br>NHDF-2	1A<br><br>1B	Retrovirus<br><br>Retrovirus	Hartfield et al. (2014)<br><br>Hartfield et al. (2014)
AH016	80	M	H1/H2	AH016-3<br>AH016-6<br>AH016-10	2A<br>2B<br>2C	Retrovirus<br>Retrovirus<br>Retrovirus	Sandor et al. (2017)<br>-<br>-
OX1 (SFC841)	36	M	H1/H2	OX1-19<br><br>SFC841-03-01<br>SFC841-03-02	3A<br><br>3B<br>3C	Retrovirus<br><br>CytoTune<br>CytoTune	van Wilgenburg et al. (2013)<br>(Dafinca et al., 2016)<br>-

Supplemental Experimental Procedures

Genotyping, sequencing and sub-cloning

Genomic DNA was extracted from cultured cells using the Illustra tissue and cells genomicPrep Mini Spin Kit (GE Healthcare, Amersham, UK). *MAPT* H1/H2 genotyping reactions were performed using AmpliTaq Gold DNA Polymerase with Gold Buffer and 2.5 mM $MgCl_2$ (Applied Biosystems), with primers to amplify around the 238 bp indel in *MAPT* intron 9 (Table S2). To determine the sequence around *MAPT* exons 9 and 10, initial amplification PCRs from genomic DNA using AmpliTaq Gold were cleaned up using FastAP (ThermoScientific) and Exonuclease I (New England BioLabs (NEB)) before a second single-primer reaction with BigDye Terminator v3.1 kit (Applied Biosystems) and sequencing on a 3730xl DNA Analyzer (Applied Biosystems) at the Zoology Sequencing Facility, University of Oxford.

Products from allele-specific amplification of the region around *MAPT* exon 10 by AmpliTaq Gold were ligated into pGEM-T Easy (Promega) and electroporated into NEB 10-beta electrocompetent *E. coli* (NEB) prior to blue-white selection on ampicillin-agar. Plasmids were isolated using the QIAprep Spin Miniprep kit (QIAGEN) and sequenced directly with BigDye.

Induced pluripotent stem cells

iPSC lines used in this manuscript are detailed in Table S1. The existing lines NHDF-1 and NHDF-2 (Hartfield et al., 2014) were used as clones 1A and 1B in this study. Further lines were generated from two MAPT H1/H2 healthy control individuals screened within the Oxford Parkinson's Disease Cohort following reprogramming either by retroviral delivery as previously described (clones 2A, 2B, 2C, 3A)(Hartfield et al., 2014; van Wilgenburg et al., 2014) or using the CytoTune-iPS Sendai Reprogramming kit (Invitrogen) (Fernandes et al., 2016) (clones 3B, 3C). Clones were adapted to feeder-free culture conditions in mTeSR™1 (StemCell Technologies), on hESC-qualified Matrigel-coated plates (BD), and routinely passaging as clumps using 0.5 mM EDTA in PBS (Beers et al., 2012). Large-scale SNP-QCed batches were frozen at p15-25 and used for experiments within a minimal number of passages post-thaw to ensure consistency.

QC analyses for previously unpublished clones are shown in Figure S1-S2, and were carried out as described previously (Hartfield et al., 2014) (Fernandes et al., 2016). FACs for pluripotency markers TRA-1-60 and Nanog (B119983, IgM-488, Biolegend; 2985S, IgG-647, Cell Signaling, with appropriate isotype controls, using the same concentration and supplier), was measured using a FACS Calibur (Becton Dickinson), and analysis using FlowJo.

Silencing of retrovirally-delivered reprogramming genes was assessed by quantitative RT-PCR as previously described (Hartfield et al., 2014). Clearance of Cytotune Sendai virus-delivered reprogramming genes was performed as previously described (Fernandes et al., 2016) by RT-PCR, run on a 1.5% agarose gel with Log2 ladder (NEB). Positive controls (fibroblasts infected 5 days previously) were always run in parallel. Primers were SeV F: GGATCACTAGGTGATATCGAGC, R: ACCAGACAAGAGTTTAAGAGATATGTATC 181bp; SOX2 F: ATGCACCGCTACGACGTGAGCGC, R: AATGTATCGAAGGTGCTCAA 451bp; KLF4 F: TTCCTGCATGCCAGAGGAGCCC, R: AATGTATCGAAGGTGCTCAA 410bp; c-MYC F:

TAACTGACTAGCAGGCTTGTCG, R: TCCACATACAGTCCTGGATGATGATG 532bp; OCT4 F: CCCGAAAGAGAAAGCGAACCAG, R: AATGTATCGAAGGTGCTCAA 483bp; β-Actin control Eurogentec 92 bp.

Assessment of conformity to pluripotent gene expression profile was performed using Illumina's Human-HT-12-v4 expression BeadChip and Pluritest (Muller et al., 2011) (pluritest.org).

Genome integrity and cell-line tracking was assessed by Illumina Human CytoSNP-12v2.1 beadchip array (~300,000 markers) or OmniExpress24 array (700,000 markers), with genomic DNA made using an All-Prep kit (Qiagen) and analysis used GenomeStudio and Karyostudio software (Illumina). The accession number for the Illumina SNP genotype and HT12v4 expression array datasets reported in this paper is GEO: GSE99125.

Differentiation of induced pluripotent stem cells to dopaminergic neuronal cultures

All cell cultures were maintained at 37ºC, 5% CO_2. Induced pluripotent stem (iPS) cells were maintained in 6-well plates coated with hESC-qualified Matrigel (Corning) with mTeSR1 medium (StemCell Technologies) changed daily. ROCK inhibitor (10 µM Y-27632) (Tocris Bioscience) was used for 24 hours after single cell passaging performed with TrypLE Express incubation (Life Technologies). iPS cells were differentiated into dopaminergic neuronal cultures according to a modified protocol of Kriks et al (Kriks et al., 2011). Prior to commencing differentiation, iPS cells were passaged as single cells and seeded in 6-well plates coated with Geltrex (Life Technologies) then grown to confluency. Basal media are as follows (Life Technologies unless stated): KO DMEM KSR = Knockout DMEM, Knockout serum replacement, 1X non-essential amino acids, 2 mM L-glutamine, 10 µM 2-mercaptoethanol (Sigma); NNB = Neurobasal medium, 0.5X N2 supplement, 0.5X B27 supplement, 2mM L-glutamine; NB = Neurobasal medium, 1X B27 supplement, 2mM L-glutamine. Differentiation factors added to basal media are as follows: 100 nM LDN-193189 (Sigma), 10 µM SB-431542 (Tocris Bioscience), 100 ng/ml recombinant sonic hedgehog C24II (R&D Systems), 2 µM purmorphamine (Calbiochem), 100 ng/ml fibroblast growth factor 8a (R&D Systems), 3 µM CHIR-99021 (Tocris Bioscience), 20 ng/ml brain-derived neurotrophic factor (Peprotech), 20 ng/ml glial cell line-derived neurotrophic factor (Peprotech), 1 ng/ml transforming growth factor β3 (Peprotech), 10 µM DAPT (abcam), 200 µM ascorbic acid (Sigma), 500 µM dibutyryl cAMP (Sigma). Medium containing differentiation and neurotrophic factors was fully changed every two days with half change every other day until day 20 of the protocol when cells were dissociated with StemPro Accutase (Life Technologies) and re-plated onto poly-L-ornithine with laminin and fibronectin or Geltrex in the desired format and density per experiment, ranging from spots of $5x10^4$ cells to an even monolayer of $3x10^5$ cells/cm^2. Cultures were treated with 1 µg/ml mitomycin C in NB medium for 1 hour to remove proliferating cells and washed with neurobasal medium before returning to fresh maturation medium. After a full medium change three days later to remove dead cells, medium was half changed every 2-3 days for the remaining period of maturation up to DIV190. For biochemical analysis of protein or RNA, cultures were washed in PBS, detached by scraping in fresh PBS, micro-centrifuged (1200 x *g*, 5 min, 4ºC), snap frozen on dry ice and stored at -80ºC until ready for lysis.

Primers used for PCR, sequencing and cloning

Primer Name	Primer Sequence (5'-3')	Primer Purpose
tau_indel_F	GGAAGACGTTCTCACTGATCTG	Genotyping H1/H2 (238 bp indel)
tau_indel_R	AAGAGTCTGGCTTCAGTCTCTC	
M13_F	GTAAAACGACGGCCAGT	Sequencing of sub-clones in pGEM-T Easy
M13_R	CAGGAAACAGCTATGAC	
MAPT_Int9seqF	TGTGAAGTGAGGACCTGCAA	Sequencing upstream of *MAPT* exon 10
MAPT_Int9seqR	AAAAGGATGAGTGACACGCC	
MAPTEx10seqF	CTCTGCCAAGTCCGAAAGTG	Sequencing *MAPT* exon 10 and environs
MAPTEx10seqR	GGTCCGTCATCTGCCCTATT	
MAPT_int9_H1F	GAAATGCAGTCGTGGGAGAC	Generation of H1 sub-clone (with MAPTEx10seqR)
MAPT_int9_H2F	TGGTTTTCTATTTCACAGCCCC	Generation of H2 sub-clone (with MAPTEx10seqR)
RBM4_F	GCCGCCATTTTAGCGTTTTG	SyBr Green qPCR of *RBM4*
RBM4_R	CACATTCCAGCACCTTCCCA	
PTBP1_F	TTGGGTCGGTTCCTGCTATT	SyBr Green qPCR of *PTBP1*
PTBP1_R	CGTCAGATCCCCGCTTTGT	

Allele-specific qRT-PCR

Specificity for the reactions was achieved with 5X SNP1 probe concentration, 3X SNP9ii probe concentration and 900 nM primers. H1:H2 ratios were calculated as follows: 2^(-1.194*[(cDNA ΔC_T H1-H2) – (Mean genomic ΔC_T H1-H2)] for SNP1 and 2^(-1.043*[(cDNA ΔC_T H1-H2) – (Mean genomic ΔC_T H1-H2)] for SNP9ii. For midbrain samples, the mean of the genomic ΔCT H1-H2 values for the eight iPS clones was used. Specific H1:H2 ratios for inclusion of exon 3 and exon 10 in MAPT transcripts were determined by dividing by the respective H1:H2 ratio for total MAPT transcripts.

TaqMan Gene Expression Assays

Gene/Assay Description	Assay ID
MAPT exon3+ transcripts (exon3-4)	Hs00902315_m1 FAM/MGB/NFQ
MAPT exon 10+ transcripts (exon9-10)	Hs00902312_m1 FAM/MGB/NFQ
MAPT all transcripts (exon12-13)	Hs00902194_m1 FAM/MGB/NFQ
GAPDH	Hs02758991_g1 VIC/MGB/NFQ
HPRT1	Hs02800695_m1 VIC/MGB/NFQ
ACTB	Hs01060665_g1 VIC/MGB/NFQ

Primers and probes for allele-specific qRT-PCR TaqMan expression assays.

Primer/Probe Name	Primer/Probe Sequence (5'-3')	Primer/Probe Purpose
MAPT exon 0 F	CCTCGCCTCTGTCGACTATC	MAPT all transcripts allele-specific (exon 0-1)
MAPT exon 1 R	TACGTCCCAGCGTGATCTTC	
MAPT exon 0 F	CCTCGCCTCTGTCGACTATC	MAPT exon 3+ transcripts allele-specific (exon 0-3)
MAPT exon 3 R	GTCACATCTTCCGCTGTTGG	
MAPT intron 0 F	CCCCAACACTCCTCAGAACT	MAPT allele-specific exon 1 genomic
MAPT exon 1 R	TACGTCCCAGCGTGATCTTC	
MAPT exon 9 F	AAGAGCCGCCTGCAGACA	MAPT exon 10+ transcripts allele-specific (exon 9-10)
MAPT exon 10 R	GGACGTTGCTAAGATCCAGCTT	
MAPT exon 9 F	AAGAGCCGCCTGCAGACA	MAPT allele-specific exon 9 genomic
MAPT intron 9 R	ACCTCCATGCACAGTCCCA	
SNP1 H1 Probe (FAM/MGB/NFQ)	CTGGTTCAAAGTTC	
SNP1 H2 Probe (VIC/MGB/NFQ)	TGGTTCAAAGCTCAC	
SNP9ii H1 Probe (FAM/MGB/NFQ)	TTGGACTTGACATTCT	
SNP9ii H2 Probe (VIC/MGB/NFQ)	CTTGGACTTGACGTTCT	

Western blotting

Cell pellets were sonicated in RIPA buffer (50 mM tris-HCl, pH 7.4, 150 mM NaCl, 1% (v/v) Triton X-100, 1% (w/v) sodium deoxycholate, 0.1% (w/v) SDS) for standard Western blotting, or in TBS (20 mM tris-HCl, pH 7.4, 140 mM NaCl) when protein dephosphorylation would subsequently be performed; cOmplete mini protease inhibitors (Roche) were added to both buffers. After 30 minutes incubation on ice, the soluble fraction was isolated by micro-centrifugation (1200 x *g*, 20 min, 4 ºC). Protein concentrations were determined by BCA assay (ThermoScientific) with a BSA calibration curve.

For protein dephosphorylation to reveal tau isoforms, 15-20 µg protein samples were incubated with lambda phosphatase (NEB) (20 units/µl, 60 min, 30ºC) followed by denaturation in Laemmli sample buffer (Laemmli, 1970) (95 ºC, 10 min). Non-dephosphorylated blots had 5 µg protein loading. Protein separation was achieved using 10% (dephosphorylated protein blots) or 4-15% (other blots) Criterion TGX polyacrylamide gels (Bio-Rad) in a tris-glycine running buffer, with transfer to a PVDF membrane using the TransBlot-Turbo Transfer System (Bio-Rad).

Blots were blocked with 5% milk (Sigma) in TBS with 0.1% (v/v) Tween 20 (TBST), incubated with primary antibody in 1% milk-TBST (overnight, 4ºC), washed three times with TBST, probed with horseradish peroxidase (HRP)-conjugated secondary antibody in 1% milk-TBST for 1 hour at room temperature, and washed four times in TBST. Immobilon Western Chemiluminescent HRP Substrate (Millipore) was added and chemiluminescence detected using the ChemiDoc Touch System (Bio-Rad). Bound proteins were removed using Restore Western Blot Stripping Buffer (ThermoScientific, 15 min) and immunodetection was repeated using additional antibodies. Primary antibodies used in this study: β3-tubulin (TUJ1) (1:1000, Covance, MMS-435P), tyrosine hydroxylase (1:2000, Millipore, ab152), HRP-conjugated β-actin (1:20,000, abcam, ab49900),

tau-1 clone PC1C6 (1:1000, Millipore, MAB3420), 4R tau repeat isoform RD4 (1:250, Millipore, 05-804), 2N tau clone 71C11 (1:1000, Covance, Sig-39408), tau-5 (1:5000, NeoMarkers, MS-247-P). Secondary antibodies used in this study: HRP-conjugated goat anti-mouse (1:5000, Bio-Rad, 170-6516), HRP-conjugated goat anti-rabbit (1:5000, Bio-Rad, 170-6515).

Immunocytochemistry

Cultures grown on glass coverslips were washed with PBS, fixed with 4% (w/v) paraformaldehyde in PBS for 10-15 min and washed three times with PBS. Cell permeabilisation and protein blocking were performed with 10% goat serum in PBS 0.1% Triton X-100 (PBST, 2 hours) prior to incubation with primary antibodies in PBST with 1% goat serum (4 ºC overnight). Coverslips were washed three times with PBST and incubated with Alexa Fluor-labelled secondary antibodies for 1 hour to enable visualisation. Coverslips were washed with PBS and incubated with 1 µg/ml DAPI (Sigma) in PBS for 5 min then washed again with PBS. Coverslips were mounted on glass slides with FluorSave (Calbiochem) and cells imaged using an EVOS FL Auto Imaging System (Life Technologies). Antibodies used in this study: TUJ1 (1:500, Covance, MMS-435P), tyrosine hydroxylase (1:250, Millipore, ab152), Alexa-488 goat anti-mouse (1:500, Invitrogen, A11001), Alexa-594 goat anti-rabbit (1:500, Invitrogen, A11012).

RNA extraction and cDNA synthesis

Midbrain samples were from healthy control subjects without any neurological symptoms obtained from the UK MRC Control collection run by Oxford Brain Bank, University of Oxford. Samples were assessed by a neuropathologist and any found to have protein deposition or morphological abnormalities except minor, age-related Alzheimer's changes were excluded from the analysis. Full ethical approval (REC15/SC/0639) and written informed consent to brain and spinal cord donation were obtained from the relatives of all potential tissue donors dying in the Oxford University Hospital Foundation Trust Hospitals.

Samples of frozen post-mortem human midbrain were obtained for *MAPT* H1/H2 individuals. Samples were composed of several cryostat-generated horizontal sections that all included the substantia nigra. Before RNA extraction, samples were homogenised in RNeasy Buffer RLT with 1% 2-mercaptoethanol using a TissueRuptor (QIAGEN).

RNA was extracted from culture cell pellets or midbrain homogenate using RNeasy Micro/Mini kits (QIAGEN) according to manufacturer's instructions. RNA concentration was determined by Nanodrop (ThermoScientific). RNA integrity number was determined for midbrain samples using a Eukaryote Total RNA Pico Assay on a 2100 Bioanalyzer System (Agilent Technologies) (n=9; mean = 6.6; median = 6.8, IQR = 1.6). First-strand cDNA synthesis was performed using SuperScript VILO MasterMix (Invitrogen) according to manufacturer's instructions with a two-hour synthesis step.

Quantitative real-time polymerase chain reaction (qRT-PCR)

All qRT-PCR assays were run as 20 µl reactions on a StepOnePlus System using either TaqMan Gene Expression Master Mix or Fast SYBR Green Master Mix (Applied Biosystems) with 200 nM of each primer. 5 ng or 10 ng cDNA was included in each reaction. TaqMan Gene Expression Assays were used to determine

expression of specific *MAPT* transcripts and multiplexed with VIC-labelled assays for housekeeping genes (Applied Biosystems, Table S3). A common threshold value of 0.1 was set for all assays (except allele-specific assays) to enable comparison. Expression of total *MAPT*, *RBM4* and *PTBP1* were determined as 2^–(Assay C_T – housekeeper geometric mean). *MAPT* isoform-specific assays were normalised to Total *MAPT* instead of housekeeper assays, with exon % inclusion determined by the formula [2^–(*MAPT* isoform assay C_T – Total *MAPT* C_T)]*100.

RNA electrophoretic mobility shift assay

SK-N-F1 cells were grown in 15 cm dishes for 48 hours and harvested by gentle scraping. The cytoplasmic fraction was first extracted using cold lysis buffer: 10 mM HEPES (Sigma), 10 mM KCl (Ambion), 0.1 mM EDTA (Ambion), 0.1 mM EGTA (Sigma), Halt protease and phosphatase inhibitor cocktails (Thermo Scientific) and 0.67 % IGEPAL CA-630 (Sigma). The nuclear pellet was washed and lysed using cold nuclear lysis buffer (20 mM HEPES, 400 mM KCl, 1 mM EDTA, 1mM EGTA and Halt protease and phosphatase inhibitor cocktails.

RNA electrophoretic mobility shift assay (EMSA) was carried out using RNA oligonucleotides biotinylated at the 3’ end, SK-N-F1 nuclear lysates and the LightShift Chemiluminescent RNA EMSA Kit (Pierce) according the manufacturer’s protocol. RNA oligonucleotides containing intron 10 WT sequence: H1 (5’ TGCATAGAATAAATCCTT**CTT**GGGCTCTCAGGA 3’) and intron 10 ΔCTT sequence (5’ TGC ATA GAA TAA ATC CTT GGG CTC TCA GGA 3’). RNA probes contain the same sequence with a 15 atom tetraethylene glycol linker biotin at the 3’ end of the oligonucleotide (Integrated DNA Technologies).

RNA-Protein pull-down

RNA pull-down of RNA binding proteins was performed using the Magnetic RNA Protein Pull-Down kit (Pierce) according to the manufacturer’s instructions. 50pmol of biotinylated RNA oligonucleotides were allowed to bind streptavidin magnetic beads and incubated with 40µg of SKNF1 nuclear enriched lysate for 1 hour at 4ºC. Unbound proteins were washed off and RNA-protein complexes were dissociated from the magnetic beads by boiling in 5X Laemmli buffer for 10 min. The isolated RNA-protein complexes were loaded onto a 10% Tris-glycine SDS-PAGE for western blotting. Blots were probed with anti-PTBP1 (1:1000, Abcam, ab30317) and anti-RBM4 (1:500, Abcam, ab130624) antibodies

Generation of lentiviral constructs and production of lentiviral particles

shRNA sequences were identified to target *MAPT* exon 10 (corresponding to 4R tau), *MAPT* exons 12-13 (constitutive exons for targeting total *MAPT*) and no known RefSeq transcript (non-targeting shRNA). Incorporation of shRNA sequences into lentiviral plasmids was performed using a modification of the PCR-based protocol of Harper and Davidson (2005), using Gibson Assembly to perform all construction steps in one (Fig. S3). The following DNA molecules were joined using Gibson Assembly Master Mix (NEB): pRRL.sin.wpre fragment of *Spe*I-HF/*Sal*I-HF double digest (NEB) of pRRL.sin.U6.shRNA.cPPT.CMV.EGFP.wpre (kind gift from Dr Óscar Cordero Llana); PCR of U6 promoter from the same construct, adding a specific shRNA sequence with the reverse primer; PCR of RRE.cPPT.pEF1α

from CSii-EF-MCS (a gift from Dr H. Miyoshi, RIKEN BioResource Center DNA Bank); PCR of EBFP2 from pBAD-EBFP2 (a gift from Robert Campbell, Addgene #14891)(Ai et al., 2007). All PCRs were performed using KAPA HiFi HotStart ReadyMix (KAPA Biosystems); see Table S5 for primer sequences. All DNA molecules were separated by agarose gel electrophoresis (2% for shRNA PCRs and 1% for all others), purified by QIAquickGel Extraction Kit (QIAGEN) and quantified by Nanodrop before Gibson Assembly. Assembly products were used to transform One Shot TOP10 Chemically Competent *E. coli* (Invitrogen) for growth and selection, then One Shot Stbl3 Chemically Competent *E. coli* (Invitrogen) following verification and sequencing.

For third generation production of lentiviral particles, the following plasmids were prepared by PureLink HiPure Plasmid Maxiprep kit (Invitrogen) and transfected into HEK293T cells in 15 cm dishes: 10 µg shRNA/EBFP2 lentiviral vector; 10 µg pMDLg/pRRE (a gift from Didier Trono, Addgene #12251)(Dull et al., 1998); 2 µg pRSV-Rev (a gift from Didier Trono, Addgene #12253); 3.4 µg pMD2.g (a gift from Didier Trono, Addgene #12259) encoding vesicular stomatitis virus glycoprotein (VSV-G) for lentiviral pseudotyping.

HEK293T cells were transfected by calcium phosphate transfection, whereby DNA was incubated with 125 mM $CaCl_2$, 25 mM HEPES, 140 mM NaCl, 0.75 mM Na_2HPO_4 (Sigma) for 30 min before adding to HEK293T cultures. On day two the culture medium was replaced, containing 10 mM sodium butyrate (Sigma), then collected eight hours later as the first harvest. Following replacement, a second harvest of medium was performed at the end of day three and pooled with the first harvest. Viral medium was centrifuged to remove debris (1,000 x *g*, 5 min), 0.45 µm filtered, then centrifuged overnight to pellet viral particles (6,000 x *g*, 4ºC). On day four the pellet was re-suspended in ice-cold PBS, ultra-centrifuged (20,000 x *g*, 90 min, 4ºC) and finally re-suspended in 20 mM Tris base, 100 mM NaCl, 10 g/l sucrose, 10 g/l D-mannitol (Sigma); brief centrifugation (1000 x *g*, 5 min, 4ºC) removed persistent debris before aliquoting for -80ºC storage.

Viral titres were determined by transduction of HEK293T cells with serial dilutions, followed by flow cytometry determination of the percentage of EBFP2-positive cells 72 hours post-transduction.

Primers for construction of lentiviral shRNA plasmids by Gibson Assembly.

Primer Name	Primer Sequence (5'-3') with shRNA sequence shown in bold	Primer Position
LenGib_1F	TTCGCCCTTACGCTCTAGAAC	Before U6
LenGib_x10R	TGTCCCTCCTCGAGAAAAAA**GGTGCAGATAATTAATAAGTTCGCTTATTAATTATCTGCACCTTC**GCAAACAAGGCTTTTCTCCAAG	To add shRNA sequence against exon 10+ *MAPT*
LenGib_TotR	TGTCCCTCCTCGAGAAAAAA**CCAGGTGGAAGTAAAATCTGATTCGTCAGATTTTACTTCCACCTGGC**GCAAACAAGGCTTTTCTCCAAG	To add shRNA sequence against total *MAPT*
LenGib_Scr	TGTCCCTCCTCGAGAAAAAA**GAAGGCTCGTCGCACTAATTTCGATTAGTGCGACGAGCCTTCTTC**GCAAACAAGGCTTTTCTCCAAG	To add scrambled shRNA sequence
LenGib_2F	TTTTTTCTCGAGGAGGGACAATTGGAGAAGTG	Before RRE
LenGib_2R	CTGCAGAATTCTCGAGACCG	After pEF1α
LenGib_3F	CGGTCTCGAGAATTCTGCAGGCCACCATGGTGAGCAAGGGCGAGG	Start of EBFP2
LenGib_3R	TGTAATCCAGAGGTTGATTGTCGACTTACTTGTACAGCTCGTCCATG	End of EBFP2

Mitochondrial axonal transport imaging

Cultures of iPS-derived dopaminergic neuronal cultures were transduced with lentiviral particles encoding shRNAs on DIV20 following re-plating onto Geltrex-coated coverslips. Cultures were imaged on a Nikon Eclipse TE-2000-U fluorescent microscope with heated chamber at 37ºC and delivery of 5% CO_2/Air for up to one hour with a 60X immersion objective (Nikon Plan APO VC, 60x/1.40 oil, 0.17, DIC, N2).

Image analysis

Kymograph time-space plots were generated from blinded videos in Fiji software (National Institutes of Health) using the Multiple Kymograph plugin and the tsp050706 macro according to its protocol (http://www.embl.de/eamnet/html/body_kymograph.html). For transduced coverslips, only EBFP2-positive axons were chosen for analysis. Within the multiple kymograph, a segmented line was drawn along the path of each mitochondrion and velocities were determined using the 'read velocities from tsp' macro. Due to the complex network that develops in extended culture, the position of the cell body was not identified so directionality could not be determined. Mitochondria were classed as motile if they moved more than 2 µm during the imaging period, corresponding to the approximate length of a mitochondrion (Vossel et al., 2015). Pauses were determined as individual parts of the path where a mitochondrion moved 0-1 pixels and/or had a velocity <0.12 pixels/s (<0.0139 µm/s) corresponding to the minimum velocity needed to reach the overall threshold of motility in the 150 s imaging window. Data are presented as average velocity (µm/s) for the measured period and also the same average velocity calculation but with pause periods removed.

Supplemental Information References

Ai, H.W., Shaner, N.C., Cheng, Z., Tsien, R.Y., and Campbell, R.E. (2007). Exploration of new chromophore structures leads to the identification of improved blue fluorescent proteins. Biochemistry *46*, 5904-5910.

Beers, J., Gulbranson, D.R., George, N., Siniscalchi, L.I., Jones, J., Thomson, J.A., and Chen, G. (2012). Passaging and colony expansion of human pluripotent stem cells by enzyme-free dissociation in chemically defined culture conditions. Nature protocols *7*, 2029-2040.

Dafinca, R., Scaber, J., Ababneh, N.a., Lalic, T., Weir, G., Christian, H., Vowles, J., Douglas, A., Fletcher-Jones, A., Browne, C., *et al.* (2016). C9orf72 Hexanucleotide Expansions are Associated with Altered ER Calcium Homeostasis and Stress Granule Formation in iPSC-Derived Neurons from Patients with Amyotrophic Lateral Sclerosis and Frontotemporal Dementia. Stem cells (Dayton, Ohio).

Dull, T., Zufferey, R., Kelly, M., Mandel, R.J., Nguyen, M., Trono, D., and Naldini, L. (1998). A third-generation lentivirus vector with a conditional packaging system. J Virol *72*, 8463-8471.

Fernandes, H.J., Hartfield, E.M., Christian, H.C., Emmanoulidou, E., Zheng, Y., Booth, H., Bogetofte, H., Lang, C., Ryan, B.J., Sardi, S.P., *et al.* (2016). ER Stress and Autophagic Perturbations Lead to Elevated Extracellular alpha-Synuclein in GBA-N370S Parkinson's iPSC-Derived Dopamine Neurons. Stem cell reports *6*, 342-356.
Harper, S.Q., and Davidson, B.L. (2005). Plasmid-based RNA interference: construction of small-hairpin RNA expression vectors. Methods Mol Biol *309*, 219-235.
Hartfield, E.M., Yamasaki-Mann, M., Ribeiro Fernandes, H.J., Vowles, J., James, W.S., Cowley, S.A., and Wade-Martins, R. (2014). Physiological characterisation of human iPS-derived dopaminergic neurons. PLoS One *9*, e87388.
Kriks, S., Shim, J.W., Piao, J., Ganat, Y.M., Wakeman, D.R., Xie, Z., Carrillo-Reid, L., Auyeung, G., Antonacci, C., Buch, A., *et al.* (2011). Dopamine neurons derived from human ES cells efficiently engraft in animal models of Parkinson's disease. Nature *480*, 547-551.
Laemmli, U.K. (1970). Cleavage of structural proteins during the assembly of the head of bacteriophage T4. Nature *227*, 680-685.
Muller, F.J., Schuldt, B.M., Williams, R., Mason, D., Altun, G., Papapetrou, E.P., Danner, S., Goldmann, J.E., Herbst, A., Schmidt, N.O., *et al.* (2011). A bioinformatic assay for pluripotency in human cells. Nature methods *8*, 315-317.
van Wilgenburg, B., Moore, M.D., James, W.S., and Cowley, S.A. (2014). The productive entry pathway of HIV-1 in macrophages is dependent on endocytosis through lipid rafts containing CD4. PLoS One *9*, e86071.
Vossel, K.A., Xu, J.C., Fomenko, V., Miyamoto, T., Suberbielle, E., Knox, J.A., Ho, K., Kim, D.H., Yu, G.Q., and Mucke, L. (2015). Tau reduction prevents Abeta-induced axonal transport deficits by blocking activation of GSK3beta. J Cell Biol *209*, 419-433.
