# Supplementary material for: MAPT Genetic Variation and Neuronal Maturity Alter Isoform Expression Affecting Axonal Transport in iPSC-Derived Dopamine Neurons
Source: Stem Cell Reports. 2017 Jul 6;9(2):587–99. doi: 10.1016/j.stemcr.2017.06.005 (PMC5549835; doi:10.1016/j.stemcr.2017.06.005)
Supplement: Document S2. Article plus Supplemental Information [file mmc2.pdf]

Stem Cell Reports
Article
ISSCR
OPEN ACCESS

MAPT Genetic Variation and Neuronal Maturity Alter Isoform Expression Affecting Axonal Transport in iPSC-Derived Dopamine Neurons

Joel E. Beevers,[1] Mang Ching Lai,[1] Emma Collins,[1] Heather D.E. Booth,[1] Federico Zambon,[1] Laura Parkkinen,[2,3] Jane Vowles,[3,4] Sally A. Cowley,[3,4] Richard Wade-Martins,[1,3,*] and Tara M. Caffrey[1,*]
[1]Department of Physiology, Anatomy and Genetics, University of Oxford, South Parks Road, Oxford OX1 3QX, UK
[2]Nuffield Department of Clinical Neurosciences, Academic Unit of Neuropathology, University of Oxford, John Radcliffe Hospital, Oxford OX3 9DU, UK
[3]The Oxford Parkinson's Disease Centre, University of Oxford, Oxford OX1 3QX, UK
[4]Sir William Dunn School of Pathology, University of Oxford, South Parks Road, Oxford OX1 3RE, UK
*Correspondence: richard.wade-martins@dpag.ox.ac.uk (R.W.-M.), tara.caffrey@dpag.ox.ac.uk (T.M.C.)
http://dx.doi.org/10.1016/j.stemcr.2017.06.005

SUMMARY

The H1 haplotype of the microtubule-associated protein tau (*MAPT*) locus is genetically associated with neurodegenerative diseases, including Parkinson's disease (PD), and affects gene expression and splicing. However, the functional impact on neurons of such expression differences has yet to be fully elucidated. Here, we employ extended maturation phases during differentiation of induced pluripotent stem cells (iPSCs) into mature dopaminergic neuronal cultures to obtain cultures expressing all six adult tau protein isoforms. After 6 months of maturation, levels of exon 3+ and exon 10+ transcripts approach those of adult brain. Mature dopaminergic neuronal cultures display haplotype differences in expression, with H1 expressing 22% higher levels of *MAPT* transcripts than H2 and H2 expressing 2-fold greater exon 3+ transcripts than H1. Furthermore, knocking down adult tau protein variants alters axonal transport velocities in mature iPSC-derived dopaminergic neuronal cultures. This work links haplotype-specific *MAPT* expression with a biologically functional outcome relevant for PD.

INTRODUCTION

Chromosome 17q21 represents an interesting genomic locus featuring an ~1.3–1.6 Mb region of linkage disequilibrium (LD) encompassing the microtubule-associated protein tau (*MAPT*) gene, including genetic variants associated with several neurodegenerative disorders. The LD surrounding *MAPT* is due to a 900 kb chromosomal inversion proposed to originate from non-allelic homologous recombination between long coding repeats flanking the region (Cruts et al., 2005), giving rise to two correspondingly large haplotype families called H1 and H2. The *MAPT* gene is of central importance to a number of neurodegenerative diseases. Strong association of *MAPT* H1 haplotype variants has been shown with progressive supranuclear palsy (PSP) (Hoglinger et al., 2011), corticobasal degeneration (CBD) (Kouri et al., 2015), and Parkinson's disease (PD) (Nalls et al., 2014).

The *MAPT* gene is characterized by diversity at the transcript and protein level. The gene expresses six transcripts through the alternative splicing of exons 2, 3, and 10 resulting in six major tau protein isoforms in the adult CNS (Andreadis et al., 1992; Goedert et al., 1989). Splicing of exons 2 and 3 yields proteins with 0, 1, or 2 N-terminal inserts (0N, 1N, and 2N tau). Exclusion or inclusion of exon 10 alters the number of microtubule binding repeats to give three or four microtubule binding repeats (3R or 4R tau). Expression of tau protein isoforms shows brain region specificity (Caffrey et al., 2006; Majounie et al., 2013; Trabzuni et al., 2012) and is regulated during development, with roles in establishing and maintaining neuronal morphology. Importantly, tau proteins have been shown to aggregate in those brain regions that degenerate in a number of diseases, collectively referred to as tauopathies. Tauopathies show differing aggregation compositions of tau protein, with principally 4R tau aggregating in PSP, CBD, and frontotemporal dementia with Parkinsonism associated with chromosome 17 (FTDP-17) (Arai et al., 2001; Buee Scherrer et al., 1996); 3R tau proteins aggregating in Pick's disease (Delacourte et al., 1996); and both 3R and 4R tau aggregating in Alzheimer's disease (Sergeant et al., 1997; Williams, 2006). Despite having a strong genetic association with *MAPT*, PD does not typically give rise to tau tangle pathologies.

While *MAPT* coding and splice site mutations have been shown to be sufficient to cause FTDP-17, the *MAPT* haplotype variants do not encode protein changes that could underlie the genetic association. We and others have previously studied haplotype effects on gene expression at the *MAPT* locus (Caffrey et al., 2006, 2008; Kwok et al., 2004; Majounie et al., 2013; Trabzuni et al., 2012). Our studies have shown that the H2 haplotype expresses twice as much exon 2+3+ *MAPT* transcript as H1 (Caffrey et al., 2008), a finding that has been replicated in a large postmortem brain series (Trabzuni et al., 2012). In addition, we showed that the *MAPT* H1 haplotype expresses 40% more exon 10+(4R) *MAPT* transcript than H2 (Caffrey et al., 2006), and this difference in exon 10+ transcript expression was greater in the globus pallidus than in the frontal cortex, demonstrating a mechanistic link between

This is an open access article under the CC BY license (http://creativecommons.org/licenses/by/4.0/).

the regulation of *MAPT* gene expression by disease-associated polymorphisms and the regional vulnerability exhibited in PSP, a 4R-tauopathy. A previous report has found an increase in the ratio of 4R:3R *MAPT* transcripts in PD brains (Tobin et al., 2008), potentially suggesting a shared mechanism of disease.

Induced pluripotent stem cell (iPSC)-derived neuronal cultures provide a powerful and tractable human neuronal model generated directly from individuals with disease, or harboring specific genetic variants. A major advantage of iPSC-derived neuronal cultures is the use in experimental studies of a key cell type of interest, enabling experimental analysis in living human neurons in a manner not attainable using postmortem tissues (Fernandes et al., 2016; Hartfield et al., 2014). Here, we differentiated dopaminergic neuronal cultures from iPSC lines heterozygous for the *MAPT* H1/H2 haplotypes to assess the effect of *MAPT* haplotype on gene expression and the role of tau protein isoforms in dopamine neurons preferentially vulnerable to degeneration in PD. iPSC-derived dopaminergic neuronal cultures express all six adult tau isoforms, approaching adult levels of expression of exon 3+ and exon 10+ transcripts after 6 months of maturation. This model was shown to be suitable to study both common genetic variations, displaying significant haplotype-specific differences in *MAPT* expression and splicing, as well as being able to characterize the effects of a rare genetic polymorphism on splicing. Finally, we perturbed the expression of both total and 4R tau in iPSC-derived dopamine neurons to demonstrate that tau isoforms regulate axonal transport velocity, linking genetic variation, gene expression, and splicing with neuronal function.

RESULTS

Establishment of Human Dopaminergic Neuronal Cultures that Express Adult Tau Isoforms

To study the relationship between genetic variation at the *MAPT* locus and PD we used human iPSCs to generate dopamine neurons from individuals carrying specific genotypes of interest. As any effect of underlying genetic polymorphic variation on gene expression and splicing is independent of disease status, we chose to use control individuals of known genotype for this study. In addition, by using individuals heterozygous for *MAPT* H1/H2, we are able to assay expression from both haplotypes in one culture controlling for confounding factors such as different genetic backgrounds, culture conditions, or environmental factors. Fifty-eight healthy controls from the Oxford Parkinson's Disease Center Discovery Cohort were screened to identify individuals heterozygous for the *MAPT* H1 and H2 alleles. Of the 58 individuals genotyped, 38% were the desired H1/H2 genotype, 53% were H1/H1, and 9% were H2/H2. Fibroblasts from H1/H2 individuals were reprogrammed to generate iPSC clones, some of which have been described previously (Dafinca et al., 2016; Hartfield et al., 2014; Sandor et al., 2017) (see also Table S1). Characterization of the new iPSC clones is presented in Figures S1 and S2. In total, eight iPSC clones made from three H1/H2 individuals were selected for use in this study (Figure 1A, see also Table S1).

Differentiation of iPSCs toward a midbrain fate generated dopaminergic neuronal cultures expressing neuron-specific beta-III tubulin (β3-tub) and the dopaminergic neuronal marker tyrosine hydroxylase (TH), identified by western blot (Figure 1B) and immunocytochemistry (Figures 1C and 1D). Approximately 65% of cells were β3-tub positive, with up to 60% of those co-expressing TH (Figure 1C).

Expression of the *MAPT* gene is both spatially and developmentally regulated, and it is primarily expressed in neurons. Adult human brain shows expression of six principal isoforms generated through the splicing of exons 2, 3, and 10 (Andreadis et al., 1992; Goedert et al., 1989), while the human fetus expresses only the shortest isoform, lacking exons 2, 3, and 10. To characterize the maturity of our dopaminergic neuronal cultures with respect to *MAPT* expression, we examined the expression of these isoforms over a 24-week (to 188 days *in vitro* [DIV188]) time course of maturation of iPSC-derived differentiation cultures using real-time qPCR. During this period the relative expression of total *MAPT* transcripts peaked at DIV48 (4 weeks after re-plating) (Figure 2A, see also Figure S3B). By maturation to DIV188, cultures exhibited a 15-fold increase in the expression of adult isoforms containing exon 3 (Figure 2B) and a 7-fold increase in the expression of adult isoforms containing exon 10 (Figure 2C), both clear evidence of neuronal maturation. Cultures maintained in culture to DIV188 reached an inclusion level of exon 3 (encoding 2N tau protein isoforms) similar to levels in postmortem human midbrain (iPSC-derived cultures, 5.6%, Figure 2B; midbrain, 6.8%, Figure 2D). At the same time point, the inclusion of exon 10 (corresponding to the 4R tau protein isoforms) had risen toward the level of inclusion measured in postmortem human midbrain (iPSC-derived cultures, 18.2%, Figure 2C; compared with midbrain at 35.2%, Figure 2D).

Dopaminergic neuronal cultures of all eight iPSC lines were matured for 6 months (DIV190) to give mature inclusion of adult-specific exons in *MAPT* transcripts. Western blot analysis of 6-month cultures revealed that the inclusion of exons 3 and 10 at the transcript level led to the presence of all six isoforms of tau protein, detected by either a pan-tau antibody or antibodies probing for 4R and 2N tau isoforms (Figure 3). Tau protein was not detected by the

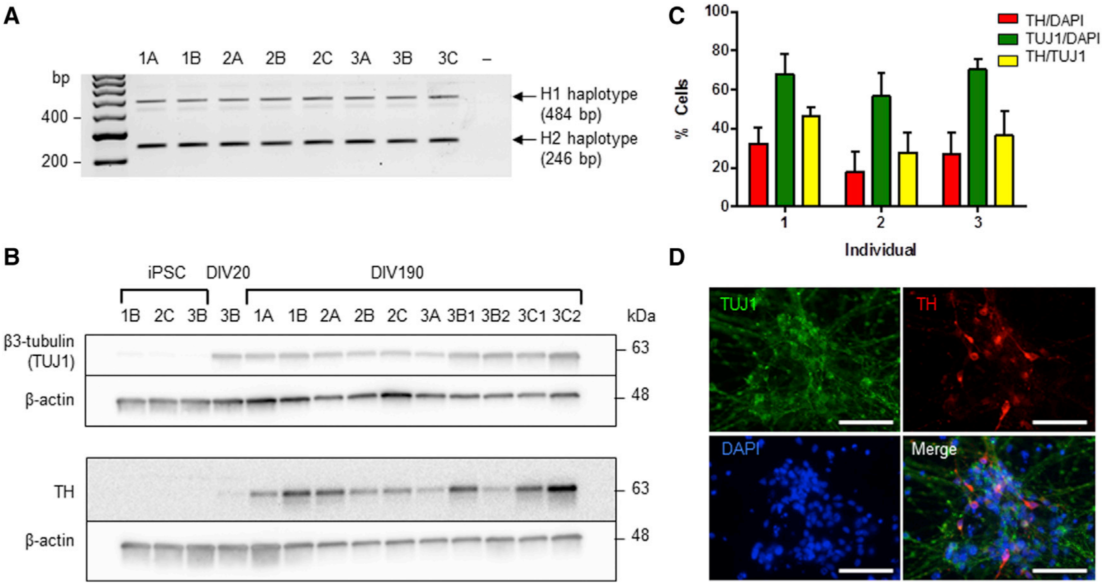

Figure 1. Differentiation of Induced Pluripotent Stem Cells with *MAPT* H1/H2 Genotype into Dopaminergic Neuronal Cultures

(A) Genotyping PCR distinguishes the 238 bp indel in *MAPT* intron 9, showing the presence of both the H1 and H2 alleles in all eight iPSC clones. Clones are identified by the number of the individual (1–3) then by the clone generated from reprogramming of the fibroblasts of that individual (A–C).

(B) Western blots showing iPSC differentiation into dopamine neuronal cultures. Neuronal marker β3-tubulin (TUJ1) (expressed by DIV20) and tyrosine hydroxylase (TH) are shown at DIV190. Samples from two differentiations are identified by a "1" or "2" suffix.

(C) Efficiency of dopaminergic differentiation was quantified from at least two clones per individual. Individuals differentiated with similar efficiencies into neurons determined by TUJ1/DAPI. The proportion of neurons that were dopaminergic was determined by TH/TUJ1. Mean ± SEM of n = 2 or 3 clones per individual; 4 images per clone.

(D) Immunofluorescent co-labelling of differentiated neuronal cultures from clone 3B fixed at DIV27 (1 week after re-plating) demonstrates expression of dopaminergic neuronal protein TH together with β3-tubulin. Scale bars, 50 μm. See also Figures S1 and S2.

antibody Tau-1 in iPSCs before commencing differentiation (Figure 3, lanes 2–4). After running this blot, one particular batch of iPSC line 1A (lane 6) was discovered to have a duplication in chromosome 1q and was removed from further analysis. Overall, these data show that, by 6 months, iPSC-derived dopaminergic neuronal cultures show significant adult-like maturity, recapitulating expression patterns seen in adult midbrain, and represent a promising model for the study of *MAPT* biology.

Haplotype-Specific Expression of *MAPT* in Human Dopaminergic Neuronal Cultures

We used our model to investigate the haplotype-specific expression of *MAPT* in dopamine neurons, the neuronal type that is preferentially vulnerable in PD. Samples from the same 6-month neuronal cultures of the eight heterozygous H1/H2 iPSC lines shown above were analyzed by real-time qPCR. The levels of total *MAPT* transcripts showed no significant difference between individuals (Figure 4A). Transcripts containing exon 3 also showed no significant difference between individuals, with the average inclusion of exon 3 for all eight lines being 9.1% (Figure 4B). However, individual 3 showed a significantly higher inclusion of exon 10 in *MAPT* transcripts (22%) compared with individuals 1 (16%) and 2 (15%) ($p = 0.0005$) (Figure 4B).

The high levels of mature *MAPT* exons 3 and 10 inclusion allow the development of allele-specific expression assays to distinguish the relative abundance of H1 and H2 transcripts using a SNP. We developed a set of TaqMan-based real-time qPCR allele-specific expression assays using SNPs in exon 1 (rs17650901) and exon 9 (rs17652121) of *MAPT* to distinguish between transcripts deriving from H1 and H2 alleles (Figure 4, see also Figures S3C–S3F). The output of these assays is the ratio of H1:H2 transcripts for a given PCR amplicon, in which a ratio greater than 1 represents more expression from the H1 allele than from the H2 allele, and a ratio less than 1 represents more expression from the H2 allele than from the H1 allele.

The expression of all *MAPT* transcripts combined, measured by amplifying transcripts between constitutive exons 0 and 1, was 23% greater from H1 than from H2 in DIV190 dopaminergic neuronal cultures (Figure 4C) ($p < 0.0001$), with significantly greater H1 expression also seen at DIV124 (11%; $p = 0.0132$) and DIV188 (31%; $p < 0.0001$)

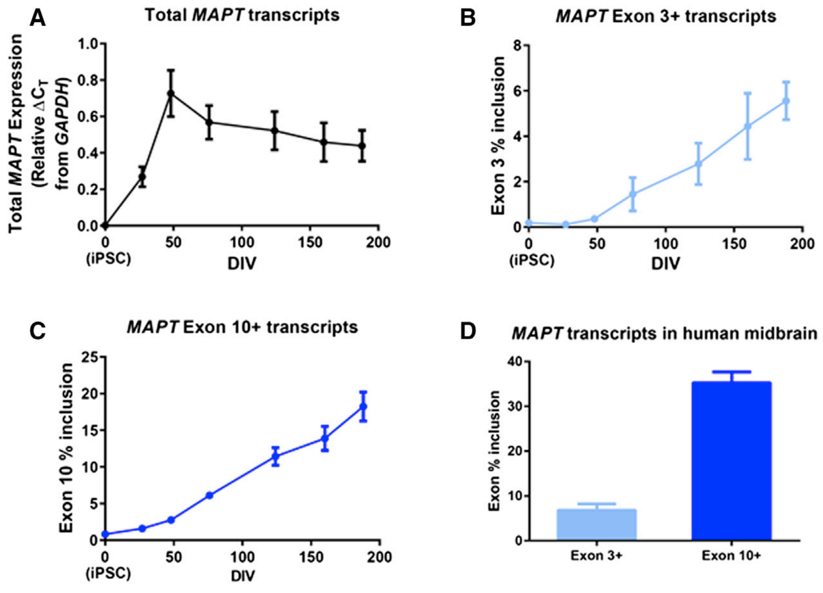

Figure 2. *MAPT* Adult Isoforms Increase in Expression over a 6-Month Differentiation

(A–C) Real-time qPCR analysis demonstrated changes in *MAPT* expression over 24 weeks of maturation to DIV188. Each graph shows mean ± SEM, n = 7 clones (all clones except 1A). At DIV188, peak mean inclusion for the alternatively spliced exons 3 and 10 was 5.6% (B) and 18.2% (C), respectively. For each graph (A–C), linear regression performed using all data points from DIV48 to DIV188 with an F test confirming that each slope is significantly different from zero: (A) $y = -0.01303 \times x + 0.7279$, $F = 4.615$, $p = 0.0391$; (B) $y = 0.2553 \times x - 0.7078$, $F = 23.32$, $p < 0.0001$; (C) $y = 0.7416 \times x - 0.05151$, $F = 96.28$, $p < 0.0001$.

(D) Exon inclusion from TaqMan-based real-time qPCR expression assays performed on human midbrain cDNA. Mean ± SEM, n = 5 individuals; exon 3+ mean = 6.79 ± 1.43; exon 10+ mean = 35.22 ± 2.43. See also Figure S3.

in the time course sample set (Figure S4). In the same assay, total *MAPT* expression in postmortem human midbrain did not differ significantly from an allelic ratio of 1 (Figure 4C).

The average H1:H2 allelic ratio of *MAPT* transcripts containing exon 3 was 0.56 across all iPSC-derived dopaminergic culture sample sets, closely matching that of 0.51 in the midbrain sample set (Figure 4D, see also Figure S4B). This demonstrates that the H2 allele expresses approximately twice as many *MAPT* transcripts containing exon 3 as the H1 allele. These exon 3 expression data from dopaminergic neuronal cultures and midbrain agree with those of postmortem brain tissue and other neuronal models studied previously (Caffrey et al., 2008; Trabzuni et al., 2012).

Finally, the allelic ratios of *MAPT* transcripts containing exon 10 were not different from 1 for DIV190 dopaminergic neuronal cultures from individuals 1 and 2 (Figure 4E). However, cultures from individual 3 showed a marked shift to a ratio of 0.51 (Figure 4E, see also Figures S4C and S4D), which we investigated further below. Consistent with previous observations in postmortem brain tissue and other neuronal models (Caffrey et al., 2006), but differing from our iPSC-derived dopaminergic neuronal cultures, postmortem human midbrain exhibited an H1:H2 ratio for exon 10+ transcripts of 1.2 (Figure 4E).

In summary, our iPSC-derived dopaminergic neuronal culture model recapitulates the allele-specific expression differences previously seen in human brain for exon 3, exhibiting a 2-fold increase from H2. Although we observed an increased total *MAPT* expression associated with the H1 *MAPT* haplotype as reported previously (Allen et al., 2014; Kwok et al., 2004), we did not observe changes in exon 10+ expression associated with *MAPT* haplotype. Interestingly, our iPSC-derived neuronal cultures permitted the observation of a further expression phenotype in the iPSC lines from individual 3.

Identification of a Genetic Variant that Alters the Inclusion of *MAPT* Exon 10

We noted that the *MAPT* exon 10 expression in dopaminergic neuronal cultures from the three iPSC clones from individual 3 was significantly different to neurons studied from five lines generated from individuals 1 and 2. Dopaminergic neuronal cultures generated from individual 3 showed an overall 40% increase in inclusion of exon 10 (Figure 4B) and a 2-fold increase in expression of exon 10+ transcripts, specifically from the H2 allele (Figure 4E). To investigate these expression phenotypes further, *MAPT* exons 9 and 10 and their flanking regions were sequenced. Individuals 1 and 2 showed the expected wild-type (WT) sequence; however, an indel was detected in individual 3 that resulted in a divergent sequence 102 bp downstream of exon 10 (Figure 5A). Primers placed in or adjacent to the haplotype-tagging 238 bp indel in intron 9 were used to specify the allele to be amplified by PCR and each chromosomal locus was subcloned and sequenced. A deletion of three nucleotides was identified in intron 10 (c.1919+102_1919+104delCTT, hereafter ΔCTT) only in the H2 allele of individual 3 and not on either allele of in individuals 1 and 2 (Figure 5B). This ΔCTT variant does not exist on public databases of genetic variation (www.ncbi.nlm.nih.gov/variation/view/), (www.ncbi.nlm.nih.gov/SNP/[Sherry et al., 2001]). As the deletion was present on the allele that showed increased exon

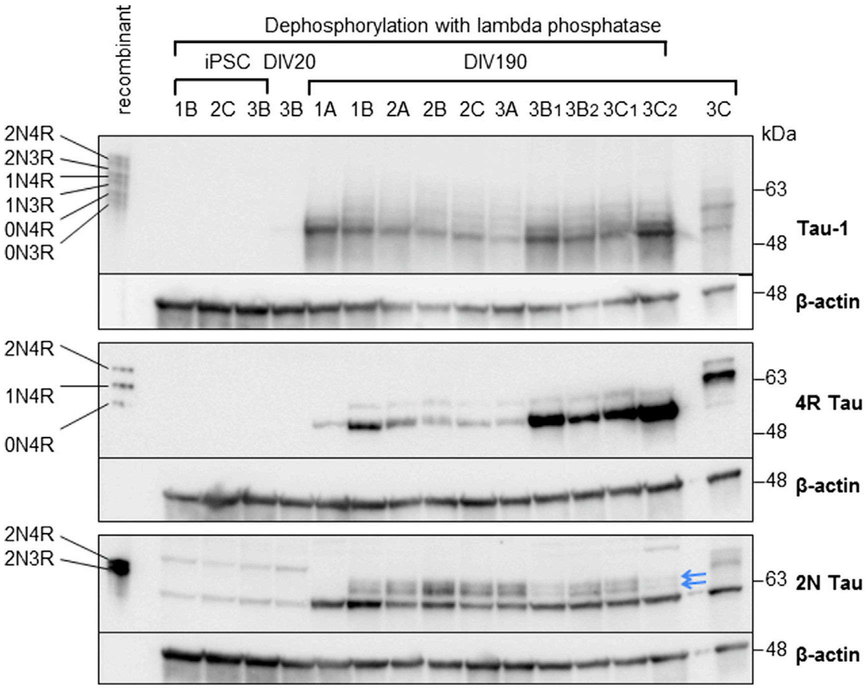

Figure 3. All Major Tau Protein Isoforms Are Expressed in iPSC-Derived Dopaminergic Neuronal Cultures Differentiated for 6 Months

Western blots showing the presence of all six major isoforms of mature tau protein in dopaminergic neuronal cultures at DIV190, as detected by antibodies against total tau (Tau-1) or specific isoforms (4R Tau, 2N Tau). All samples dephosphorylated, except the right lane as an untreated control. 2N Tau blot: central bands indicated by blue arrows represent the two 2N Tau isoforms. Additional bands are considered non-specific as they appear in the iPSC lysate in which no tau is detectable.

10 inclusion, this strongly suggests that the ΔCTT variant causes increased inclusion of exon 10 in agreement with the altered H1:H2 exon 10 inclusion ratio.

To examine possible functional mechanisms for the increased inclusion of exon 10 in transcripts that include the ΔCTT variant, an *in silico* search of splice factor binding sites was performed on the intronic sequence with or without the ΔCTT variant using *SpliceAid 2* (Figure 5, see also Figure S5) (Piva et al., 2012). Searching for all available splicing factors, two binding sites were predicted to be lost by the ΔCTT variant: RBM4, which is predicted to enhance inclusion of exon 10 (Kar et al., 2006), and PTBP1 (PTB/hnRNP I), which is predicted to promote exclusion of exon 10 (Wang et al., 2004). In the presence of the ΔCTT variant, the predicted binding site for RBM4 would be reduced from 9 to 6 bp, and that of PTBP1 from 6 to only 3 bp. Real-time qPCR for *RBM4* and *PTBP1* confirmed the expression of both of these splicing factors in cultures of clones from each of the three individuals across the period of the time course (Figures S5C and S5D). Furthermore, the expression of *RBM4* and *PTBP1* appears to be correlated in these samples (Figure S5E). From DIV48, the expression levels of both genes appears to be relatively stable, rather than matching the observed increase in inclusion of exon 10 over time. Importantly, although there was a clear difference in *MAPT* exon 10 inclusion between individual 3 and individuals 1 and 2 (Figure S5F), there was no difference in the expression of *RBM4* and *PTBP1* in the clone from individual 3 when compared with clones from individuals 1 and 2, showing that there is no intrinsic difference in the expression of these splicing factors that could otherwise explain the phenotype of individual 3.

We performed RNA-electrophoretic mobility shift assays to study the impact of the ΔCTT sequence on RNA-protein complex formation between biotinylated RNA probes containing the exon 10 WT or ΔCTT sequence and SK-N-F1 nuclear protein extract (Figure 5C). Four RNA-protein complexes (I–IV) were formed using the WT RNA probe (Figure 5C, lane 2), while only three (II–IV) were visible when the CTT sequence was deleted (Figure 5C, lane 8), indicating that the CTT deletion reduced the number of species of protein complexes interacting with the RNA. The band shift intensities of the WT sequence and nuclear extract were much stronger than those observed for the ΔCTT sequence (Figure 5C, lanes 2 and 8), suggesting that the CTT sequence forms part of an important RNA motif for protein binding. We further assessed the binding strengths of the WT and ΔCTT sequences by competition experiments in which unlabeled RNA oligonucleotides competed with the probes for complex formation (Figure 5C, lanes 3–6, 9–12). The ΔCTT competitor showed a reduced competition strength compared with the WT competitor when assayed with the WT probe for complex formation (Figure 5C, lanes 3–6), whereas the two competitor oligonucleotide sequences exhibited comparable competition strength with the ΔCTT probe (Figure 5C, lanes 9–12).

We probed for interaction of PTBP1 and RBM4 after pull-down of nuclear proteins with the WT or ΔCTT probes

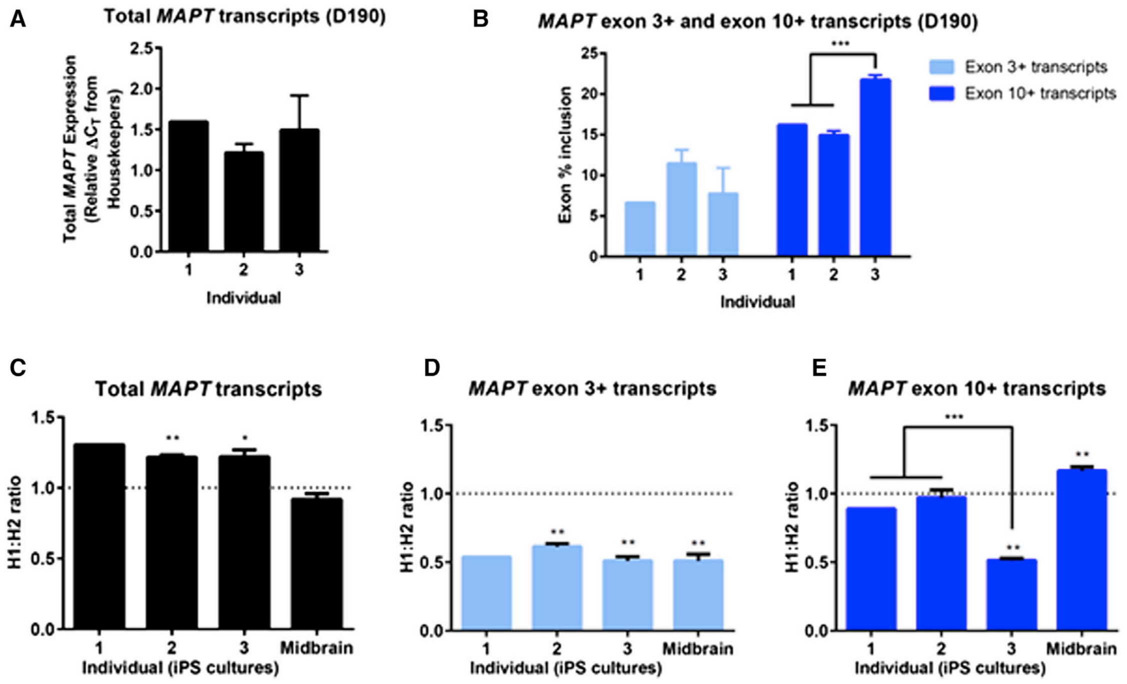

Figure 4. Dopaminergic Neuronal Cultures Exhibit Significant Differences in Isoform Expression from H1 and H2 Alleles at 6 Months

TaqMan-based real-time qPCR expression assays on samples at DIV190. Mean ± SEM; individual 1, n = 1 clone; individuals 2 and 3, n = 3 clones; ≥3 cDNA samples per clone.

(A) Total *MAPT* expression reported as relative ΔC_T of geometric mean of three housekeeper genes (*GAPDH, HPRT1,* and *ACTB*). n.s., not significant, one-way ANOVA.

(B) Percent inclusion of alternatively spliced exons 3 (light blue) and 10 (dark blue) at DIV190. The three individuals show similar inclusion of exon 3, whereas individual 3 shows a significantly greater inclusion of exon 10. Significant difference between groups in an unpaired t test: ***p = 0.0005.

(C–E) Allele-specific expression assays distinguishing transcripts of H1 and H2 allelic origin, presented as H1:H2 ratio, i.e., values >1 show higher H1 expression. Data from analysis of human midbrain (C) n = 9; (D) n = 5; and (E) n = 9. (C) Individuals 2 and 3 show significantly greater expression of total *MAPT* transcripts from the H1 chromosome (individual 2, **p = 0.0059; individual 3, *p = 0.0471; midbrain, n.s.). (D and E) The H1:H2 ratio is normalized to the H1:H2 ratio of total *MAPT* transcripts per sample. *Significant difference from mean of 1 in a one-sample t test. (D) There are 2-fold greater exon 3-containing transcripts coming from the H2 chromosome. Individual 2, **p = 0.0032; individual 3, **p = 0.0040; midbrain, **p = 0.0020. (E) Haplotype-specific expression of exon 10 varies between individuals and midbrain: Individual 2, n.s.; individual 3, ***p = 0.0008; midbrain, **p = 0.0005. Significant difference between groups in an unpaired t test: ***p = 0.0005. See also Figures S3 and S4.

(Figure 5D). Firstly, this technique successfully shows an interaction of PTPB1 with both probes, whereas the signal for RBM4 rose barely above background despite robust presence in the starting lysate. Secondly, the PTBP1 interaction with the ΔCTT variant is greatly reduced compared with the WT sequence. The results indicate that a CTT deletion in this region could potentially reduce the binding of RNA binding proteins such as PTPB1, thereby altering the balance of exon 10 inclusion and exclusion in transcripts.

Tau Knockdown Alters Velocity of Axonal Transport in Human Dopaminergic Neuronal Cultures

The *MAPT* H1 allele is established to carry risk for PD, and we describe above an increased allelic expression of total *MAPT* transcripts in dopaminergic neuronal cultures compared with the protective H2 allele (Figure 4C). Furthermore, our postmortem human midbrain samples confirmed increased expression of exon 10+ *MAPT* transcripts from the H1 allele (Figure 4E). We note that both of these scenarios would contribute to having more molecules of 4R tau protein present in cells carrying the H1 allele. We therefore developed a dual approach to investigate the functional effect of 4R tau expression in dopaminergic neuronal cultures. We hypothesized that a decrease in the level of total tau expression, or 4R tau expression, both of which mimic the protective H2 condition, may be beneficial to dopamine neurons. We designed short hairpin RNAs (shRNAs) to target either a constitutive

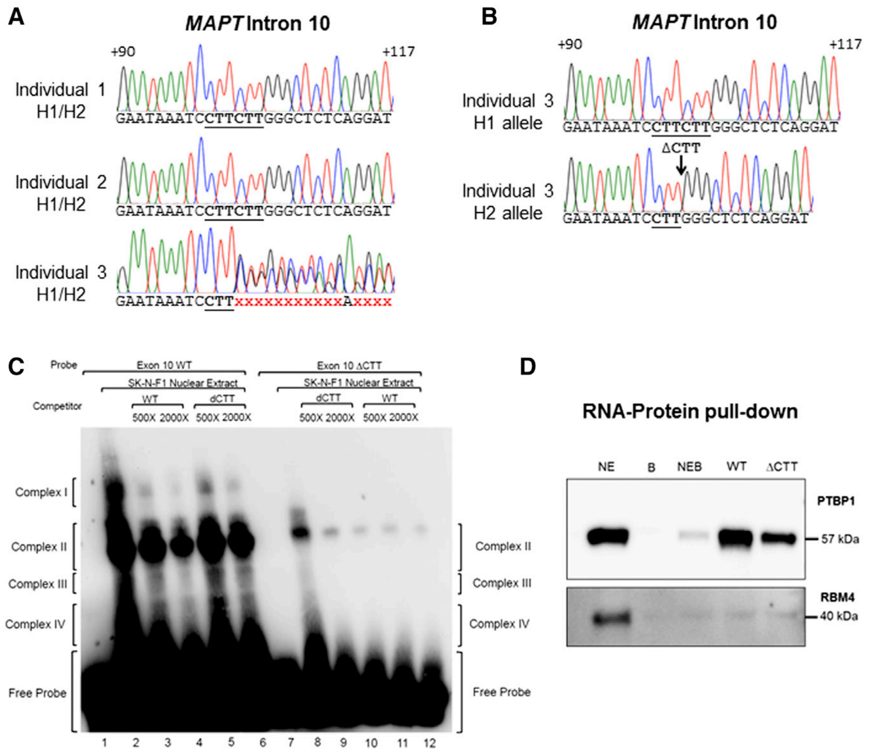

Figure 5. A Deletion in *MAPT* Intron 10 Decreases Binding of Factors that Regulate Exon 10 Splicing

(A) Sequencing chromatograms for *MAPT* intron 10 showing the expected sequence for genomic DNA (both H1 and H2 alleles together) for individuals 1 and 2. The red x's represent divergent chromatograms from two overlapping sequences caused by an indel on one allele for individual 3.

(B) Sequencing chromatograms for *MAPT* intron 10 showing single allelic genomic DNA from individual 3. The H2 allele showed a ΔCTT variation.

(C) Electrophoretic mobility shift assay using an RNA probe for an intron 10 wild-type (WT) sequence (lanes 1–6) and an RNA probe for an intron 10 ΔCTT variant sequence (lanes 7–12), with and without competitors.

(D) Western blot of RNA-protein pull-down using the WT and ΔCTT RNA probes. Lanes indicate proteins obtained from nuclear extract only (NE), beads only (B), NE and B (NEB), WT RNA probe and the ΔCTT probe. Blots are shown for PTPB1 and RMB4. See also Figure S5.

exon of *MAPT* (i.e., all transcripts), or targeted specifically at *MAPT* transcripts including exon 10, or a scrambled shRNA not matching any known RefSeq transcript. These three shRNA sequences were incorporated into lentiviral plasmids. Following transduction at DIV20 shortly after re-plating, dopaminergic neuronal cultures were matured for either 4 weeks or 5 months. Tau protein was knocked down by 65% at 4 weeks (Figure 6A), with knockdown persisting at 5 months post-transduction (Figure 6B), while maintaining expression of the blue fluorescent protein (EBFP2) reporter (Figure 6C). The shRNA targeting *MAPT* exon 10 efficiently knocked down 4R tau protein in 5-month cultures (Figure 6B). Cultures were incubated with MitoTracker Deep Red to enable visualization of mitochondria for live fluorescence imaging (Figure 6D). After using the EBFP2 reporter to identify transduced neurons, analysis of kymographs generated from time-lapse imaging enabled measurement of the velocity of mitochondria (Figure 6E).

When compared with the scrambled shRNA control, there was no significant difference in mitochondrial velocity when tau protein was reduced in young neurons (4 weeks post-transduction; Figure 6F, upper). However, in older neurons (5 months post-transduction) where the complement of tau proteins is more representative of expression in the adult human brain, knockdown by the 4R *MAPT* shRNA caused a significant increase in median mitochondrial velocity from 0.148 to 0.574 μm/s (adjusted $p = 0.0007$; Figure 6F, lower left). Furthermore, when the period during which a mitochondrion has paused is removed from the calculations for average velocity, both knockdown conditions (4R and total *MAPT*) produced a significant increase in median mitochondrial velocity compared with the scrambled shRNA control, from 0.272 μm/s for scrambled to 0.793 and 0.519 μm/s for 4R and total *MAPT* knockdown, respectively (4R *MAPT* adjusted $p = 0.0002$; total *MAPT* adjusted $p = 0.0191$; Figure 6F, lower right). As the level of total tau protein is similar between the two knockdown conditions, these data suggest that the disruption of the balance of 4R and 3R tau isoforms is what is giving rise to the most significant differences in mitochondrial velocity. This increase remains, and is reinforced, after removal of pause periods, suggesting that the alteration in velocity is not simply due to a reduction in the chance of a mitochondrion stalling/pausing, but to an intrinsic increase in the velocity of axonal transport.

Due to the requirement for high-density cultures to generate TH+ dopaminergic neurons, it was not possible to determine transport direction in our assay system as axons were not able to be assigned to specific cell bodies. Kinesin produces movement solely in the anterograde direction, whereas cytoplasmic dynein, while predominantly a retrograde motor, is capable of reversing and so functions bidirectionally (Dixit et al., 2008), so we therefore stratified the data by separating mitochondria that had >90% of their

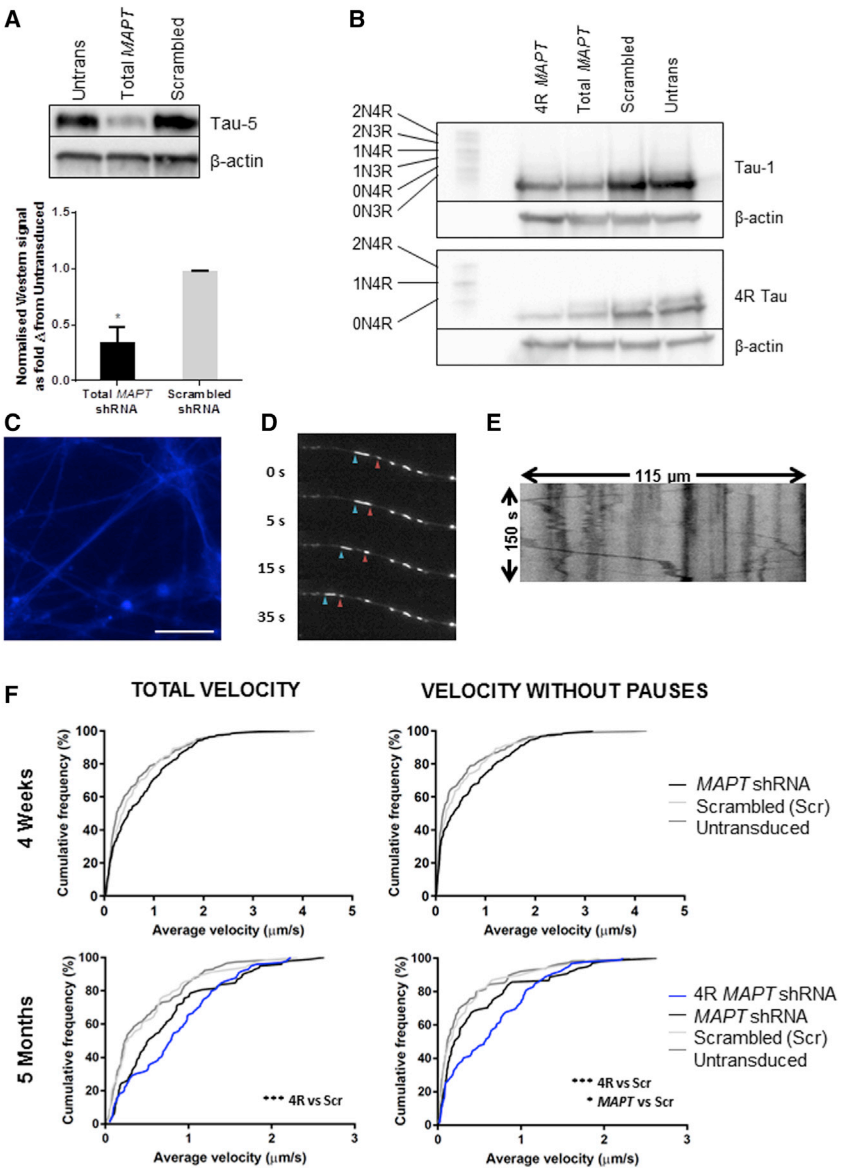

Figure 6. Knockdown of 4R Tau Increases Velocity of Mitochondrial Axonal Transport in iPSC-Derived Dopaminergic Neuronal Cultures Differentiated for 5 Months

(A) Western blots showing knockdown of tau protein in dopaminergic neuronal cultures 4 weeks post-transduction. $^{*}p = 0.0457$.

(B) Western blots after protein dephosphorylation show knockdown of tau protein and 4R tau isoforms persisting in 5-month dopaminergic neuronal cultures with β-actin loading control.

(C) Fluorescence microscopy image of EBFP2 expression during axonal transport live imaging at 5 months. Scale bar, 25 μm.

(D) Selected time-lapse fluorescence microscopy images of axonal mitochondria in dopaminergic neuronal cultures stained with MitoTracker Deep Red. Arrowheads show motile mitochondria.

(E) Kymograph time-space plot of a trace along the linear path of an axon, from which mitochondrial motility parameters can be determined.

(F) Cumulative frequency graphs of average mitochondrial velocity in dopaminergic neuronal cultures at (top) 4 weeks post-transduction and (bottom) 5 months post-transduction. (Left) Average total velocity of each measured motile mitochondrion; (right) average velocity of each measured motile mitochondrion after removal of time when paused. All four graphs were statistically significant in Kruskall-Wallis nonparametric tests: upper left, $p = 0.0033$; upper right, $p = 0.0200$; lower left, $p < 0.0001$; lower right, $p < 0.0001$. *Significance in the follow-up Dunn's multiple comparisons test (nonparametric) compared with scrambled shRNA control: $^{*}p < 0.05$; $^{***}p < 0.001$. See also Figure S6.

movement in a single direction (unidirectional) from those that were bidirectional. In young neurons, no significant difference was observed between knockdown and scrambled conditions. In older neurons, knockdown of 4R tau resulted in a significant increase in velocity for mitochondria moving in one direction only, with or without inclusion of paused periods (Figure S6E: $p = 0.0048$; Figure S6F, $p = 0.0033$), but showed no difference in velocity of bidirectional mitochondria (Figures S6G and S6H), showing that the overall effect seen by 4R tau knockdown was carried by unidirectional mitochondria. In contrast, knockdown of total tau resulted in increased velocity of both unidirectional and bidirectional mitochondria, but only when pause periods were removed (Figure S6F, $p = 0.0078$; Figure S6H $p = 0.0327$). These data suggest tau regulates kinesin anterograde transport to a greater extent than dynein anterograde transport.

DISCUSSION

In this work, we have established an iPSC-derived dopaminergic neuronal cell culture model suitable for the study of the expression and function of *MAPT* in those neurons preferentially vulnerable to neurodegeneration in PD. We have shown that iPSC-derived dopaminergic neuronal cultures expressing a high level of the neuronal markers β3-tub and TH show increasing expression of adult tau

isoforms throughout the maturation phase. Notably, levels of expression of exon 3+ and exon 10+ transcripts approach adult levels after 6 months of neuronal maturation, with strong expression of 2N and 4R tau protein isoforms evident. This highly physiological *MAPT* expression profile makes iPSC-derived dopaminergic neurons a highly suitable system to study the genetic regulation of tau expression in a tractable human dopamine neuronal culture model.

An important aspect of tau biology is the effect of *MAPT* genetic variation on the expression of tau isoforms. Discovery of exon 10 splice site mutations in pedigrees with FTDP-17 demonstrated for the first time that *MAPT* noncoding genetic variation changed the balanced expression of the 3R and 4R tau protein, and that the imbalanced expression was sufficient to cause disease (Hutton et al., 1998; Spillantini et al., 1998). Studies by our group (Caffrey et al., 2006, 2008) and others (Majounie et al., 2013; Trabzuni et al., 2012) have demonstrated that common *MAPT* genetic variation associated with disease alters the expression of tau transcripts and that this effect varies between brain regions. Combining these attributes of tau biology requires tau to be studied in a tractable model expressing the adult tau isoforms, such as iPSC-derived dopaminergic neuronal cultures.

Within the field of stem cell models of neurodegeneration, the age and maturity of iPSC-derived neuronal cultures is central to developing better models to study disease. *MAPT* isoform expression is developmentally regulated and therefore gives an excellent genetic marker of neuronal maturity. iPSC-derived dopaminergic neuronal cultures differentiated and matured using the protocol that we use here (Kriks et al., 2011) show increasingly more adult tau isoform expression over a maturation period of 6 months, with full-length 2N4R tau protein being detected at DIV190. Other studies of dopaminergic cultures detected mostly fetal tau present after 20 days in maturation medium (Ehrlich et al., 2015), which is in line with our findings. Similar expression patterns are noted in iPSC-derived cortical neuronal cultures. A detailed examination of tau proteins by mass spectrometry provided confirmation of the predominance of 0N3R tau peptides and the lack of peptides corresponding to exons 2 and 3 in 5-week iPSC-derived cortical neurons (Silva et al., 2016). Wray and colleagues (Sposito et al., 2015) noted that their iPSC-derived cortical cultures go through the developmental switch from fetal to adult tau after 365 days. Alteration of the developmental switch to adult tau isoforms occurs in cultures with tau mutations in which increased expression of 4R tau is observed at earlier time points than control (Ehrlich et al., 2015; Iovino et al., 2015; Silva et al., 2016; Sposito et al., 2015). Despite differences in cortical and dopaminergic differentiation protocols, it is clear that the expression of the adult isoforms of tau requires extended periods of maturation. This is perhaps unsurprising considering the natural developmental switch of expression from fetal tau to adult tau over time in the human brain. As the tau isoform balance has proven key to the pathogenicity of splice site mutations in FTDP-17, and is a characteristic of the genetic association, the expression of the adult isoforms in neuronal cell culture models is vitally important for future tau biology work.

Our differentiated neuronal cultures allow us to investigate the effect of common genetic variation on *MAPT* expression. The allele-specific expression assays we developed detect differences in expression from the H1 and H2 haplotypes within heterozygous lines. We demonstrate that the dopaminergic neuronal cultures express 22% greater tau transcripts from the H1 chromosome than H2. This allelic difference in total tau expression has not been demonstrated in studies of postmortem tissue (Caffrey et al., 2006; Trabzuni et al., 2012), although other groups have reported it using genetic reporter constructs (Kwok et al., 2004). Whether this difference would change with even longer maturation phases is as yet unclear; however, we observe an increasing H1:H2 total tau over our time course. Interestingly, Hayesmoore et al. (2009) noted a negative correlation between H1:H2 total transcript ratio and age, indicating that age-related changes in haplotype expression are relevant in the human brain. When we examine the alternatively spliced *MAPT* transcripts at 6 months maturation, neurons possess a mature expression profile, recapitulating the 2-fold greater exon 3 containing transcripts from the H2 chromosome correctly mirroring expression in midbrain and other postmortem tissue regions (Caffrey et al., 2008; Trabzuni et al., 2012).

Our iPSC-derived dopamine neuronal cultures express exon 10 from the H1 and H2 chromosomes at equal ratio which differs from the ratio observed here in the midbrain tissue, as well as in previous publications of postmortem brain tissue (Caffrey et al., 2006; Majounie et al., 2013). It is possible this discrepancy arises due to the maturity or age of the culture as we document an increase in the expression of tau isoforms over extended maturation phases, although inclusion does not reach the 35% level seen in the midbrain samples. While the exon 10-containing transcripts maintain a similar H1:H2 transcript ratio throughout, it is possible that the ratios could change as exon 10 inclusion approaches the level of the adult midbrain. Alternatively, while this iPSC-derived model of dopaminergic neurons cultures provides a system in which to assay the function of the adult tau isoforms, it is possible that the culture system here may not support all the factors to recapitulate exon 10 inclusion as seen in the brain, which in turn would explain why the adult levels of exon 10 expression have not been achieved.

In our study we identified a rare non-coding variant within the *MAPT* locus. The previously undescribed ΔCTT variant occurring within intron 10 was found to alter the inclusion of exon 10 in transcripts and to change the balance of 4R tau protein isoforms. We observed a greater number of protein complexes bound the WT sequence than the ΔCTT variant, which agrees with in silico analysis predictions of the loss of two binding sites for RBM4 and PTBP1. We suggest that a partial loss of binding of RBM4 and a near abrogation of binding of PTBP1 would increase the likelihood of inclusion of exon 10 in transcripts originating from the ΔCTT H2 allele; alternatively, the loss of binding of both factors could alter the balance of remaining factors to promote exon 10 inclusion. The ability to assay the effect of genetic variation at the expression level is the first step in studying a range of tauopathies, from diseases caused by specific tau mutations such as those found in FTDP-17, to diseases with strong genetic associations with *MAPT* as in PD, PSP, and CBD.

It is clear from work on FTDP-17 splice site mutations (Hutton et al., 1998; Spillantini et al., 1998), as well as investigations into haplotype-specific expression of tau isoforms (Caffrey et al., 2006, 2008; Trabzuni et al., 2012), that the balance of tau isoform expression plays a major role in disease; however, the functional impact of subtle changes in expression brought about by common variants has yet to be fully investigated. The importance of the expression of alternative tau isoforms in the study of axonal transport was previously suggested by a study investigating the regulation of dynein and kinesin motor proteins. The longest tau isoform (2N4R) was shown to be a less potent inhibitor of both kinesin and dynein than the shortest tau isoform (0N3R) (Dixit et al., 2008). The high levels of adult tau isoform expression in our differentiated neuronal cultures allows us to investigate the effect of tau isoform expression on neuronal function. We have shown that depletion of 4R tau isoforms leads to increased axonal transport velocity in 5-month-old dopaminergic neuronal cultures. As this effect is strengthened after removal of pause periods it is likely that the alteration in velocity is not simply due to a reduction in the chance of a mitochondrion stalling/pausing, but to an intrinsic increase in the velocity of axonal transport. In addition, the effect of knockdown was greatest in mitochondria with unidirectional movement, which may support the data that indicate that tau affects kinesin anterograde movement greater than dynein (Dixit et al., 2008). We propose that the H2 haplotype, which expresses reduced 4R tau compared with the H1 haplotype, may exert a protective effect as it allows for more fluid mitochondrial movement along axons with high energy requirements, such as the dopaminergic neurons that degenerate in PD.

Advances in iPSC-derived neuronal models have opened up a new frontier for the study of human neurons as highly relevant genetic models, capturing the variants present in the donor. iPSC-derived dopaminergic neuronal cultures are known to be of high neurophysiological relevance (Hartfield et al., 2014) and reveal cellular phenotypes when comparing patients and controls (Fernandes et al., 2016) suitable for target and drug discovery. Here, we have been able to exploit iPSC-derived neurons to model the genetic basis of susceptibly to common disease by specifically choosing certain genotypes to functionally determine the effect of genetic variants on cell biology in the specific cell type of interest. Together, these attributes of iPSC-derived neuronal models demonstrate the great potential of studying neurons in a dish for future investigation into the genetic basis of neurodegenerative disorders.

EXPERIMENTAL PROCEDURES

iPSCs

All iPSC lines were derived from dermal fibroblasts from disease-free donors recruited through the Oxford Parkinson's Disease Center: participants were recruited to this study having given signed informed consent, which included derivation of hiPSC lines from skin biopsies (Ethics Committee: National Health Service, Health Research Authority, NRES Committee South Central, Berkshire, UK, who specifically approved this part of the study [REC 10/H0505/71]). All iPSC lines are detailed in Supplemental Experimental Procedures, Figures S1 and S2; Table S1.

Differentiation of iPSCs to Dopaminergic Neuronal Cultures

iPSCs were differentiated into dopaminergic neuronal cultures according to a modified protocol of Kriks et al. (2011) (see Supplemental Experimental Procedures). The medium was half changed every 2–3 days for the extended periods of maturation up to DIV190.

Allele-Specific Real-Time qPCR

Pairs of TaqMan probes were identified to distinguish the H1 and H2 alleles at *MAPT* SNP1 (rs17650901) and SNP9ii (rs17652121) Myers et al. (2007), and ordered as custom probe-only assays from Applied Biosystems. Validation of specificity was performed using H1/H1 or H2/H2 genomic DNA, followed by the generation of standard curves from 8:1 to 1:8 with *MAPT* H1 and H2 BAC constructs (Figure S3), which were adjusted through the origin.

Mitochondrial Axonal Transport Imaging

Cultures of iPSC-derived dopaminergic neuronal cultures were transduced with lentiviral particles encoding shRNAs on DIV20. Cultures were maintained until 4 weeks post re-plating or 5 months post re-plating before imaging. Cultures were incubated with MitoTracker Deep Red (Invitrogen) for 30 min then washed with Hank's

balanced salt solution with calcium and magnesium. Time-lapse imaging with Cy5 channel was performed using Volocity software to generate 150 images at 1-s intervals with 110-ms exposure (Vossel et al., 2015). Single EBFP2 images were taken on the DAPI channel to identify transduced neurons for each video.

Statistical Analysis

All statistical analysis and graphical representation was performed in Prism version 7 (GraphPad software). Specific tests are noted in each figure legend. For allele-specific expression studies, two-tailed one-sample t tests were used to determine whether the mean H1:H2 ratio was significantly different from 1. Unpaired two-tailed t tests were used to compare the expression levels of two groups. For axonal transport data, motile mitochondria from multiple imaging sessions were combined to produce a cumulative frequency (%) plot and analyzed by Kruskall-Wallis test with Dunn's multiple-testing correction. Knockdown conditions were compared with non-targeted shRNA conditions.

SUPPLEMENTAL INFORMATION

Supplemental Information includes Supplemental Experimental Procedures, six figures, and one table and can be found with this article online at http://dx.doi.org/10.1016/j.stemcr.2017.06.005.

AUTHOR CONTRIBUTIONS

J.E.B. designed and performed experiments, analysed data, and wrote the manuscript. M.C.L. and E.C. performed experiments. H.E.D.B. and F.Z. assisted in iPSC-derived differentiations and protocol development. L.P. provided post-mortem brain tissue. J.V. and S.A.C. generated and characterized the iPSCs. R.W.M. and T.M.C. funded the project, provided the concept, designed and interpreted experiments, and wrote the manuscript.

ACKNOWLEDGMENTS

The work was supported by the Monument Trust Discovery Award from Parkinson's UK. J.E.B. was supported by a Studentship from Parkinson's UK (H-1102). T.M.C. was supported by funds from IMI StemBANCC (no. 115439). We thank O. Cordero Llana for technical assistance. The research leading to these results has received support from the Innovative Medicines Initiative Joint Undertaking under grant agreement no. 115439, resources of which are composed of financial contributions from the European Union's Seventh Framework Program (FP7/2007–2013) and EFPIA companies. This publication reflects only the authors' views, and the IMI JU, EFPIA, and the European Commission are not liable for any use that may be made of the information contained therein. The Wellcome Trust (WTISSF121302) and The Oxford Martin School (LC0910-004) provide financial support to the James Martin Stem Cell Facility (to S.A.C.). We thank the High-Throughput Genomics Group at the Wellcome Trust Center for Human Genetics, Oxford (funded by Wellcome Trust grant reference 090532/Z/09/Z and MRC Hub grant G0900747 91070) for the generation of Illumina data. We acknowledge the Oxford Brain Bank, supported by the Medical Research Council (MRC), Brains for Dementia Research (BDR) (Alzheimer Society and Alzheimer Research UK), Autistica UK, and the NIHR Oxford Biomedical Research Center.

Received: December 6, 2016
Revised: June 6, 2017
Accepted: June 6, 2017
Published: July 6, 2017

REFERENCES

Allen, M., Kachadoorian, M., Quicksall, Z., Zou, F., Chai, H.S., Younkin, C., Crook, J.E., Pankratz, V.S., Carrasquillo, M.M., Krishnan, S., et al. (2014). Association of MAPT haplotypes with Alzheimer's disease risk and MAPT brain gene expression levels. Alzheimers Res. Ther. *6*, 39.

Andreadis, A., Brown, W.M., and Kosik, K.S. (1992). Structure and novel exons of the human tau gene. Biochemistry *31*, 10626–10633.

Arai, T., Ikeda, K., Akiyama, H., Shikamoto, Y., Tsuchiya, K., Yagishita, S., Beach, T., Rogers, J., Schwab, C., and McGeer, P.L. (2001). Distinct isoforms of tau aggregated in neurons and glial cells in brains of patients with Pick's disease, corticobasal degeneration and progressive supranuclear palsy. Acta Neuropathol. *101*, 167–173.

Buee Scherrer, V., Hof, P.R., Buee, L., Leveugle, B., Vermersch, P., Perl, D.P., Olanow, C.W., and Delacourte, A. (1996). Hyperphosphorylated tau proteins differentiate corticobasal degeneration and Pick's disease. Acta Neuropathol. *91*, 351–359.

Caffrey, T.M., Joachim, C., Paracchini, S., Esiri, M.M., and Wade-Martins, R. (2006). Haplotype-specific expression of exon 10 at the human MAPT locus. Hum. Mol. Genet. *15*, 3529–3537.

Caffrey, T.M., Joachim, C., and Wade-Martins, R. (2008). Haplotype-specific expression of the N-terminal exons 2 and 3 at the human MAPT locus. Neurobiol. Aging *29*, 1923–1929.

Cruts, M., Rademakers, R., Gijselinck, I., van der Zee, J., Dermaut, B., de Pooter, T., de Rijk, P., Del-Favero, J., and van Broeckhoven, C. (2005). Genomic architecture of human 17q21 linked to frontotemporal dementia uncovers a highly homologous family of low-copy repeats in the tau region. Hum. Mol. Genet. *14*, 1753–1762.

Dafinca, R., Scaber, J., Ababneh, N., Lalic, T., Weir, G., Christian, H., Vowles, J., Douglas, A.G., Fletcher-Jones, A., Browne, C., et al. (2016). C9orf72 hexanucleotide expansions are associated with altered endoplasmic reticulum calcium homeostasis and stress granule formation in induced pluripotent stem cell-derived neurons from patients with amyotrophic lateral sclerosis and frontotemporal dementia. Stem Cells *34*, 2063–2078.

Delacourte, A., Robitaille, Y., Sergeant, N., Buee, L., Hof, P.R., Wattez, A., Laroche-Cholette, A., Mathieu, J., Chagnon, P., and Gauvreau, D. (1996). Specific pathological Tau protein variants characterize Pick's disease. J. Neuropathol. Exp. Neurol. *55*, 159–168.

Dixit, R., Ross, J.L., Goldman, Y.E., and Holzbaur, E.L. (2008). Differential regulation of dynein and kinesin motor proteins by tau. Science *319*, 1086–1089.

Ehrlich, M., Hallmann, A.L., Reinhardt, P., Arauzo-Bravo, M.J., Korr, S., Ropke, A., Psathaki, O.E., Ehling, P., Meuth, S.G., Oblak, A.L., et al. (2015). Distinct neurodegenerative changes in an induced pluripotent stem cell model of frontotemporal dementia linked to mutant TAU protein. Stem Cell Reports *5*, 83–96.

Fernandes, H.J., Hartfield, E.M., Christian, H.C., Emmanoulidou, E., Zheng, Y., Booth, H., Bogetofte, H., Lang, C., Ryan, B.J., Sardi, S.P., et al. (2016). ER stress and autophagic perturbations lead to elevated extracellular alpha-synuclein in GBA-N370S Parkinson's iPSC-derived dopamine neurons. Stem Cell Reports *6*, 342–356.

Goedert, M., Spillantini, M.G., Jakes, R., Rutherford, D., and Crowther, R.A. (1989). Multiple isoforms of human microtubule-associated protein tau: sequences and localization in neurofibrillary tangles of Alzheimer's disease. Neuron *3*, 519–526.

Hartfield, E.M., Yamasaki-Mann, M., Ribeiro Fernandes, H.J., Vowles, J., James, W.S., Cowley, S.A., and Wade-Martins, R. (2014). Physiological characterisation of human iPS-derived dopaminergic neurons. PLoS One *9*, e87388.

Hayesmoore, J.B., Bray, N.J., Cross, W.C., Owen, M.J., O'Donovan, M.C., and Morris, H.R. (2009). The effect of age and the H1c MAPT haplotype on MAPT expression in human brain. Neurobiol. Aging *30*, 1652–1656.

Hoglinger, G.U., Melhem, N.M., Dickson, D.W., Sleiman, P.M., Wang, L.S., Klei, L., Rademakers, R., de Silva, R., Litvan, I., Riley, D.E., et al. (2011). Identification of common variants influencing risk of the tauopathy progressive supranuclear palsy. Nat. Genet. *43*, 699–705.

Hutton, M., Lendon, C.L., Rizzu, P., Baker, M., Froelich, S., Houlden, H., Pickering-Brown, S., Chakraverty, S., Isaacs, A., Grover, A., et al. (1998). Association of missense and 5'-splice-site mutations in tau with the inherited dementia FTDP-17. Nature *393*, 702–705.

Iovino, M., Agathou, S., Gonzalez-Rueda, A., Del Castillo Velasco-Herrera, M., Borroni, B., Alberici, A., Lynch, T., O'Dowd, S., Geti, I., Gaffney, D., et al. (2015). Early maturation and distinct tau pathology in induced pluripotent stem cell-derived neurons from patients with MAPT mutations. Brain *138*, 3345–3359.

Kar, A., Havlioglu, N., Tarn, W.Y., and Wu, J.Y. (2006). RBM4 interacts with an intronic element and stimulates tau exon 10 inclusion. J. Biol. Chem. *281*, 24479–24488.

Kouri, N., Ross, O.A., Dombroski, B., Younkin, C.S., Serie, D.J., Soto-Ortolaza, A., Baker, M., Finch, N.C., Yoon, H., Kim, J., et al. (2015). Genome-wide association study of corticobasal degeneration identifies risk variants shared with progressive supranuclear palsy. Nat. Commun. *6*, 7247.

Kriks, S., Shim, J.W., Piao, J., Ganat, Y.M., Wakeman, D.R., Xie, Z., Carrillo-Reid, L., Auyeung, G., Antonacci, C., Buch, A., et al. (2011). Dopamine neurons derived from human ES cells efficiently engraft in animal models of Parkinson's disease. Nature *480*, 547–551.

Kwok, J.B., Teber, E.T., Loy, C., Hallupp, M., Nicholson, G., Mellick, G.D., Buchanan, D.D., Silburn, P.A., and Schofield, P.R. (2004). Tau haplotypes regulate transcription and are associated with Parkinson's disease. Ann. Neurol. *55*, 329–334.

Majounie, E., Cross, W., Newsway, V., Dillman, A., Vandrovcova, J., Morris, C.M., Nalls, M.A., Ferrucci, L., Owen, M.J., O'Donovan, M.C., et al. (2013). Variation in tau isoform expression in different brain regions and disease states. Neurobiol. Aging *34*, 1922.e7–1922.e12.

Myers, A.J., Pittman, A.M., Zhao, A.S., Rohrer, K., Kaleem, M., Marlowe, L., Lees, A., Leung, D., McKeith, I.G., Perry, R.H., et al. (2007). The MAPT H1c risk haplotype is associated with increased expression of tau and especially of 4 repeat containing transcripts. Neurobiol. Dis. *25*, 561–570.

Nalls, M.A., Pankratz, N., Lill, C.M., Do, C.B., Hernandez, D.G., Saad, M., DeStefano, A.L., Kara, E., Bras, J., Sharma, M., et al. (2014). Large-scale meta-analysis of genome-wide association data identifies six new risk loci for Parkinson's disease. Nat. Genet. *46*, 989–993.

Piva, F., Giulietti, M., Burini, A.B., and Principato, G. (2012). SpliceAid 2: a database of human splicing factors expression data and RNA target motifs. Hum. Mutat. *33*, 81–85.

Sandor, C., Robertson, P., Lang, C., Heger, A., Booth, H., Vowles, J., Witty, L., Bowden, R., Hu, M., Cowley, S.A., et al. (2017). Transcriptomic profiling of purified patient-derived dopamine neurons identifies convergent perturbations and therapeutics for Parkinson's disease. Hum. Mol. Genet. *26*, 552–566.

Sergeant, N., David, J.P., Lefranc, D., Vermersch, P., Wattez, A., and Delacourte, A. (1997). Different distribution of phosphorylated tau protein isoforms in Alzheimer's and Pick's diseases. FEBS Lett. *412*, 578–582.

Sherry, S.T., Ward, M.H., Kholodov, M., Baker, J., Phan, L., Smigielski, E.M., and Sirotkin, K. (2001). dbSNP: the NCBI database of genetic variation. Nucleic Acids Res. *29*, 308–311.

Silva, M.C., Cheng, C., Mair, W., Almeida, S., Fong, H., Biswas, M.H., Zhang, Z., Huang, Y., Temple, S., Coppola, G., et al. (2016). Human iPSC-derived neuronal model of tau-A152T frontotemporal dementia reveals tau-mediated mechanisms of neuronal vulnerability. Stem Cell Reports *7*, 325–340.

Spillantini, M.G., Murrell, J.R., Goedert, M., Farlow, M.R., Klug, A., and Ghetti, B. (1998). Mutation in the tau gene in familial multiple system tauopathy with presenile dementia. Proc. Natl. Acad. Sci. USA *95*, 7737–7741.

Sposito, T., Preza, E., Mahoney, C.J., Seto-Salvia, N., Ryan, N.S., Morris, H.R., Arber, C., Devine, M.J., Houlden, H., Warner, T.T., et al. (2015). Developmental regulation of tau splicing is disrupted in stem cell-derived neurons from frontotemporal dementia patients with the 10 + 16 splice-site mutation in MAPT. Hum. Mol. Genet. *24*, 5260–5269.

Tobin, J.E., Latourelle, J.C., Lew, M.F., Klein, C., Suchowersky, O., Shill, H.A., Golbe, L.I., Mark, M.H., Growdon, J.H., Wooten, G.F., et al. (2008). Haplotypes and gene expression implicate the MAPT region for Parkinson disease: the GenePD study. Neurology *71*, 28–34.

Trabzuni, D., Wray, S., Vandrovcova, J., Ramasamy, A., Walker, R., Smith, C., Luk, C., Gibbs, J.R., Dillman, A., Hernandez, D.G., et al. (2012). MAPT expression and splicing is differentially regulated by

brain region: relation to genotype and implication for tauopathies. Hum. Mol. Genet. *21*, 4094–4103.

Vossel, K.A., Xu, J.C., Fomenko, V., Miyamoto, T., Suberbielle, E., Knox, J.A., Ho, K., Kim, D.H., Yu, G.Q., and Mucke, L. (2015). Tau reduction prevents Abeta-induced axonal transport deficits by blocking activation of GSK3beta. J. Cell Biol. *209*, 419–433.

Wang, J., Gao, Q.S., Wang, Y., Lafyatis, R., Stamm, S., and Andreadis, A. (2004). Tau exon 10, whose missplicing causes frontotemporal dementia, is regulated by an intricate interplay of *cis* elements and *trans* factors. J. Neurochem. *88*, 1078–1090.

Williams, D.R. (2006). Tauopathies: classification and clinical update on neurodegenerative diseases associated with microtubule-associated protein tau. Intern. Med. J. *36*, 652–660.

Stem Cell Reports, Volume *9*

Supplemental Information

MAPT Genetic Variation and Neuronal Maturity Alter Isoform Expression Affecting Axonal Transport in iPSC-Derived Dopamine Neurons

Joel E. Beevers, Mang Ching Lai, Emma Collins, Heather D.E. Booth, Federico Zambon, Laura Parkkinen, Jane Vowles, Sally A. Cowley, Richard Wade-Martins, and Tara M. Caffrey

Supplemental Information

Figure S1

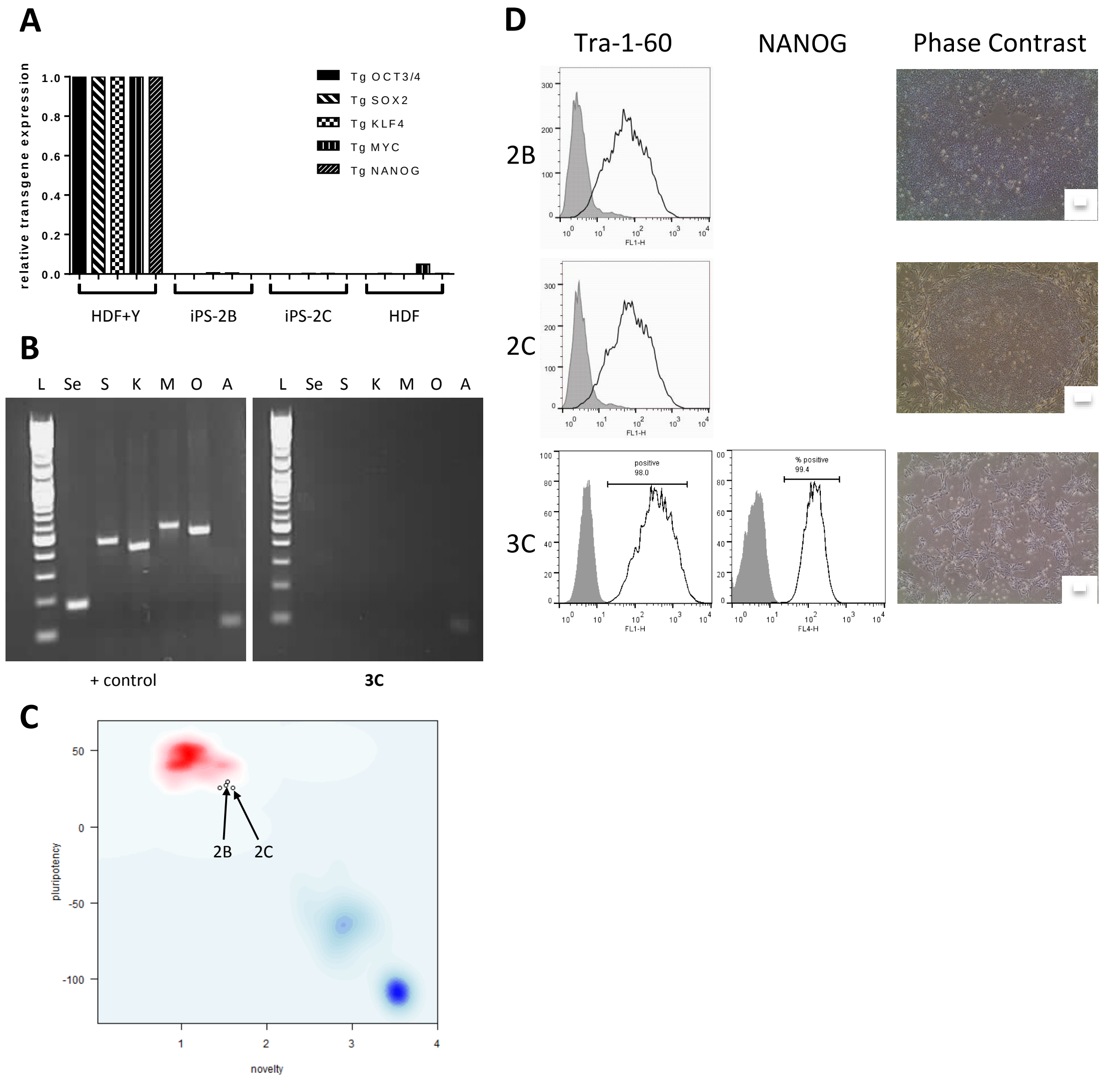

Figure S1: Characterisation of previously unpublished iPSC clones 2B, 2C and 3C, related to Experimental Procedures and Figure 1.

(A) Retroviral transgene silencing in iPSC lines 2B and 2C by qRT-PCR for each transgene, normalised to actin endogenous control, relative to expression of transgenes from fibroblasts 5 days post-infection with the Yamanaka reprogramming retroviruses (HDF+Y), with uninfected fibroblasts (HDF) as a negative control.

(B) CytoTune Sendai virus clearance shows the correct size band for β-actin, and no bands corresponding to the reprogramming virus RT-PCR product sizes for iPSC line 3C; L, Log2 ladder; Se, Sendai backbone 181 bp; S, Sox2 451 bp; K, Klf4 410 bp; M, c-myc 532 bp; O, Oct-4 483 bp; A, β-actin control 92 bp; + control, fibroblasts infected with CytoTune 5 days previously.

(C) PluriTest analysis of Illumina HT12v4 transcriptome array data shows the tested iPSC lines cluster in the red cloud representing pluripotent cell lines and not with differentiated cells (blue clouds).

(D) iPSC lines express expected pluripotency proteins as shown by FACs for Tra-1-60 and Nanog (grown feeder-free); open black plot represents antibody, filled grey plot is isotype control; Right-hand panel shows the expected iPSC colony morphology, with high nucleus to cytoplasm ratio by phase-contrast microscopy; 2B is photographed on matrigel, 2C on feeders, 3C photographed one day after thawing onto matrigel, so not yet clustered tightly together. Scale bar = 100 µm.

Figure S2

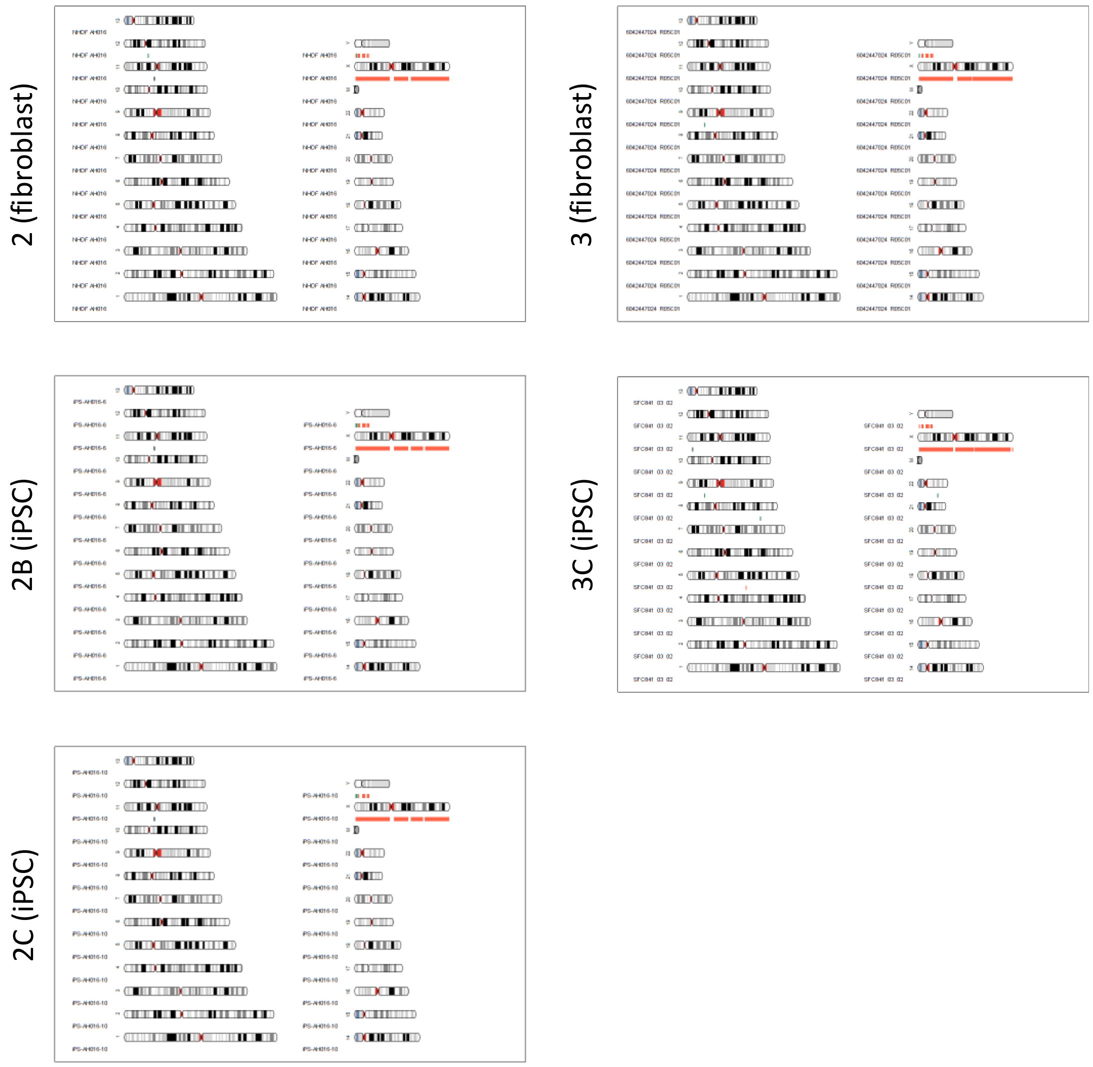

Figure S2: Further characterisation of previously unpublished iPSC clones 2B, 2C and 3C, related to Experimental Procedures and Figure 1.

Genome integrity was assessed by Illumina Human CytoSNP-12v2.1 or OmniExpress24 SNP array and karyograms produced using KaryoStudio software (Illumina); autosome amplifications (green), deletions (orange) and LOH regions (grey) are shown alongside the relevant chromosome; single-copy sex chromosomes are annotated orange, X chromosomes for females are grey.

Figure S3

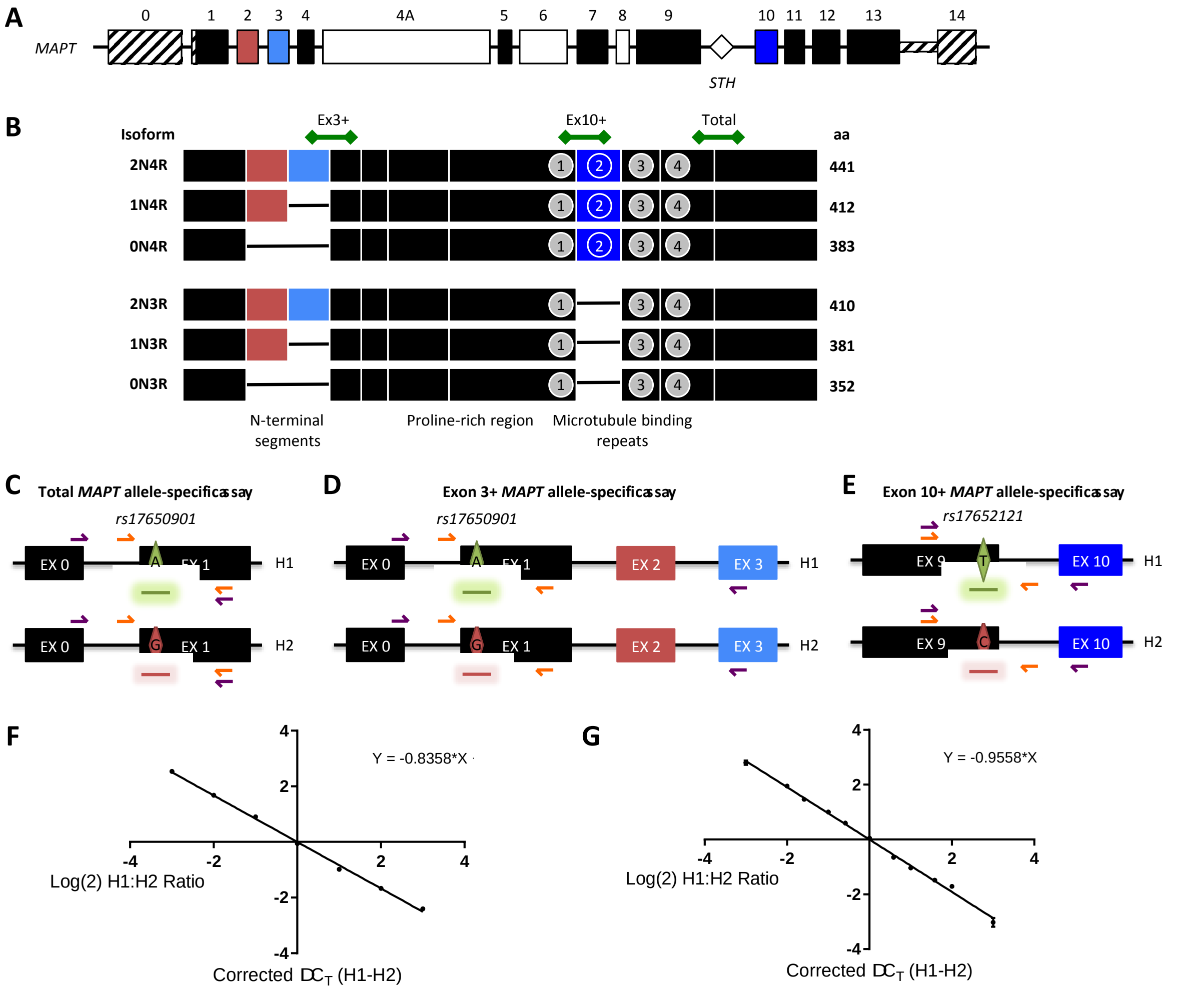

Figure S3: Schematics of the human *MAPT* locus and quantitative RT-PCR assays, related to Figures 2 and 4.

(A) The human *MAPT* locus on chromosome 17q21.31 contains 16 exons; exons 2, 3 and 10 (coloured) are subject to alternative splicing, exons 9-12 encode microtubule-binding domains with a high degree of sequence similarity and exons 4A, 6 and 8 (white) are not expressed in the adult central nervous system.

(B) Adult human brain expresses six major isoforms of tau protein through the alternative splicing of exons 2, 3 and 10. The locations of the standard (not allele-specific) TaqMan-based quantitative RT-PCR expression assays used in this study are shown in green.

(C-E) Schematics of the allele-specific TaqMan-based expression assays exploiting haplotype-tagging SNPs to distinguish the allelic origin of transcripts, with the H1 or H2 allele shown respectively as a green or red diamond. TaqMan probes bearing FAM (green) or VIC (red) are shown as coloured lines below the SNP. Arrows denote primers to amplify from genomic DNA (orange) or cDNA (purple). (C) Allele-specific assay for total *MAPT* transcripts using SNP rs17650901 (SNP1) in exon 1. (D) Allele-specific assay for exon 3+ *MAPT* transcripts also using SNP rs17650901 (SNP1) in exon 1. (E) Allele-specific assay for exon 10+ *MAPT* transcripts using SNP rs17652121 in exon 9 (SNP9ii).

(F-G) Standard curves for (F) the allele-specific expression assay using SNP1 (rs17650901) and (G) the allele-specific expression assay using SNP9ii (rs17652121). Standard curves were generated using *MAPT* H1 PAC and *MAPT* H2 PAC in ratios from 8:1 to 1:8. The values of ΔC_T (H1-H2) were plotted against the log(2) H1:H2 ratio, then corrected to remove the value of the y-intercept (to bring the graph through the origin).

Figure S4

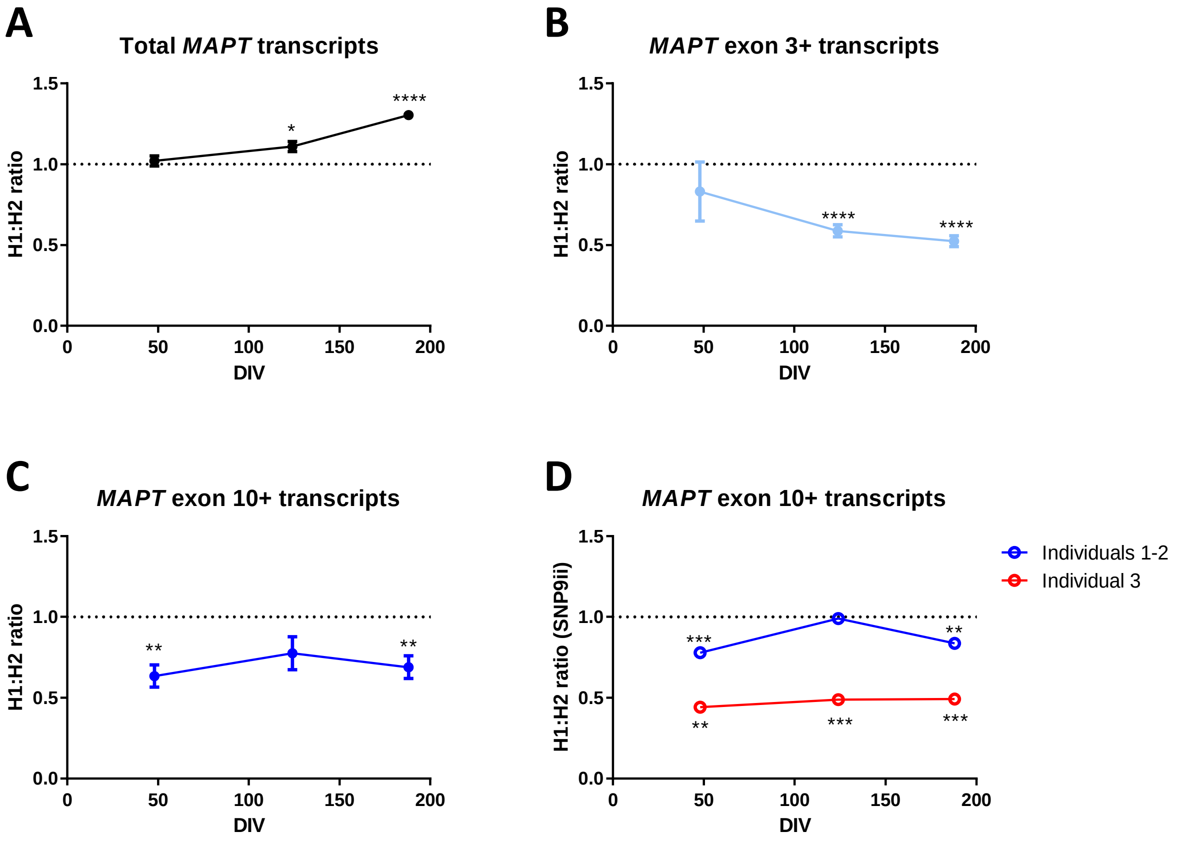

Figure S4: Allele-specific *MAPT* expression assays on dopaminergic neuronal cultures over 24-week maturation, related to Figure 4.

(A-C) Data from allele-specific TaqMan-based quantitative RT-PCR expression assays on samples from three points of a time course of maturation of dopaminergic neuronal cultures: DIV48, DIV124 and DIV188. Each graph shows mean ±SEM, n=7 clones (all clones except clone 1A). Asterisks denote significant statistical difference from hypothetical mean of 1 in a one-sample *t*-test: (A) DIV48, mean=1.020 ± 0.031, n.s.; DIV124, mean=1.109 ± 0.031, p=0.0132, t=3.478, df=6; DIV188, mean=1.305 ± 0.024, p<0.0001, t=12.58, df=6; (B) DIV48, mean=0.832 ± 0.183, n.s.; DIV124, mean=0.588 ± 0.038, p<0.0001, t=10.97, df=6; DIV188, mean=0.524 ± 0.034, p<0.0001, t=13.95, df=6; (C) DIV48, mean=0.635 ± 0.069, p=0.0019, t=5.280, df=6; DIV124, mean=0.776 ± 0.103, n.s.; DIV188, mean=0.689 ± 0.070, p=0.0044, t=4.437, df=6.

(D) Subdivision of graph C to separate out individual 3, which consistently showed a distinct phenotype relative to exon 10. Individuals 1-2, n=4 clones; individual 3, n=3 clones. Asterisks denote significant statistical difference from hypothetical mean of 1 in a one-sample *t*-test: individuals 1-2 DIV48, mean=0.780 ± 0.013, p=0.0005, t=16.50, df=3; individuals 1-2 DIV124, mean=0.991 ± 0.030, n.s.; individuals 1-2 DIV188, mean=0.837 ± 0.014, p=0.0013, t=11.74, df=3; individual 3 DIV48, mean=0.441 ± 0.019, p=0.0011, t=29.85, df=2; individual 3 DIV124, mean=0.488 ± 0.006, p=0.0002, t=81.41, df=2; individual 3 DIV188, mean=0.492 ± 0.006, p=0.0002, t=81.39, df=2.

Figure S5

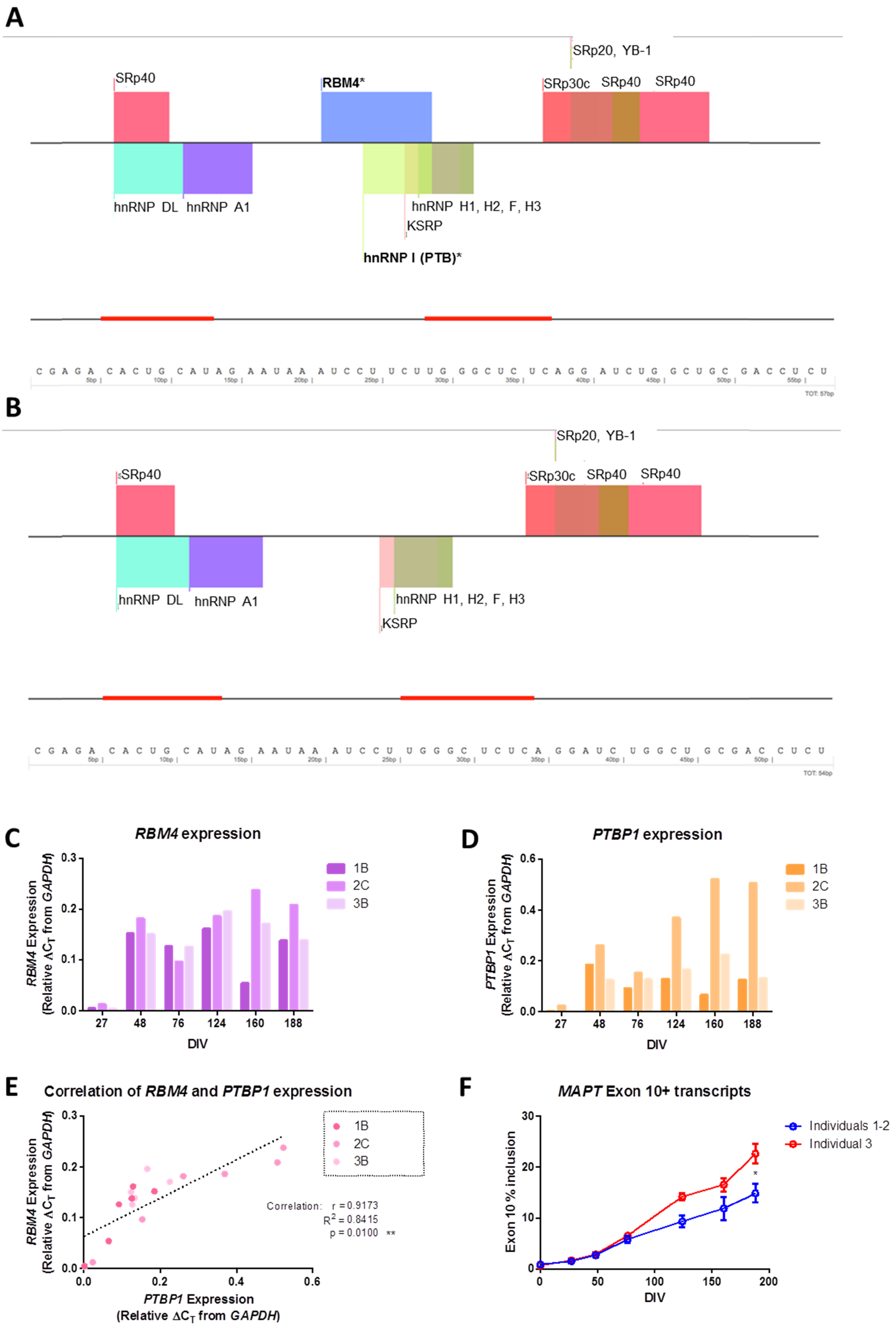
A
SRp20, YB-1
SRp40
RBM4*
SRp30c
SRp40
SRp40
hnRNP DL
hnRNP A1
hnRNP H1, H2, F, H3
KSRP
hnRNP I (PTB)*
C G A G A C A C U G C A U A G A A U A A A U C C U U C U U G G G C U C U C A G G A U C U G G C U G C G A C C U C U
B
SRp20, YB-1
SRp40
SRp30c
SRp40
SRp40
hnRNP DL
hnRNP A1
hnRNP H1, H2, F, H3
KSRP
C G A G A C A C U G C A U A G A A U A A A U C C U U G G G C U C U C A G G A U C U G G C U G C G A C C U C U
C
RBM4 expression
RBM4 Expression (Relative ΔCT from GAPDH)
1B
2C
3B
DIV
27
48
76
124
160
188
D
PTBP1 expression
PTBP1 Expression (Relative ΔCT from GAPDH)
1B
2C
3B
DIV
E
Correlation of RBM4 and PTBP1 expression
RBM4 Expression (Relative ΔCT from GAPDH)
PTBP1 Expression (Relative ΔCT from GAPDH)
1B
2C
3B
Correlation: r = 0.9173
R2 = 0.8415
p = 0.0100 **
F
MAPT Exon 10+ transcripts
Exon 10 % inclusion
DIV
Individuals 1-2
Individual 3

Figure S5: Splice factor binding site and expression analysis, related to Figure 5. Screen shots of splice factor binding analysis with *Splice Aid 2* online tool (http://193.206.120.249/splicing_tissue.html, version February 2013, 71 splicing factors, 2339 RNA binding sites, accessed February 2016) (Piva *et al.* 2012) using an input of **(A)** a 57 bp portion of the consensus sequence of *MAPT* intron 10 and **(B)** the equivalent 54 bp ΔCTT sequence as in control 3. Splice factors that promote exon inclusion or exclusion are shown as blocks respectively above or below the central line. Unlike the consensus sequence, the ΔCTT variant sequence is not predicted to be bound by RBM4 (blue box above) or hnRNP I (PTB; light green box below). All known splicing factors were included in this search, rather than restricting by tissue type.

(C-F) Quantitative RT-PCR expression over the time course of maturation for dopaminergic neuronal cultures from DIV27 to DIV188. **(C-D)** qRT-PCR expression data for *RBM4* and *PTBP1* (PTB/hnRNP I). Both splice factors are significantly expressed at the RNA level from at least DIV48 onwards but show no correlation with the rising inclusion of exon 10 (see part D). No significant difference is observed for control 3. Bars represent mean of three technical values of n=1 cDNA sample, with each of the three individuals (one clone per individual) represented as a separate bar.

(E) Alternative representation of the data from graphs A-B to reveal correlation of the expression of *RBM4* and *PTBP1* transcripts across the time course; statistical results for Pearson correlation are presented on the graph.

(F) qRT-PCR expression data for *MAPT* exon 10+ transcripts, representing a subdivision of Fig. 2C to separate out individual 3 bearing the ΔCTT variant. Individuals 1-2, n=4 clones; individual 3, n=3 clones. Asterisk denotes significant statistical difference between groups in a multiple one-sample *t*-test with Sidak-Bonferroni multiple comparison correction: $mean_{1\text{-}2}=14.91 \pm 1.85$, $mean_3=22.66 \pm 1.98$, p=0.000133, t=4.36, df=31

Figure S6

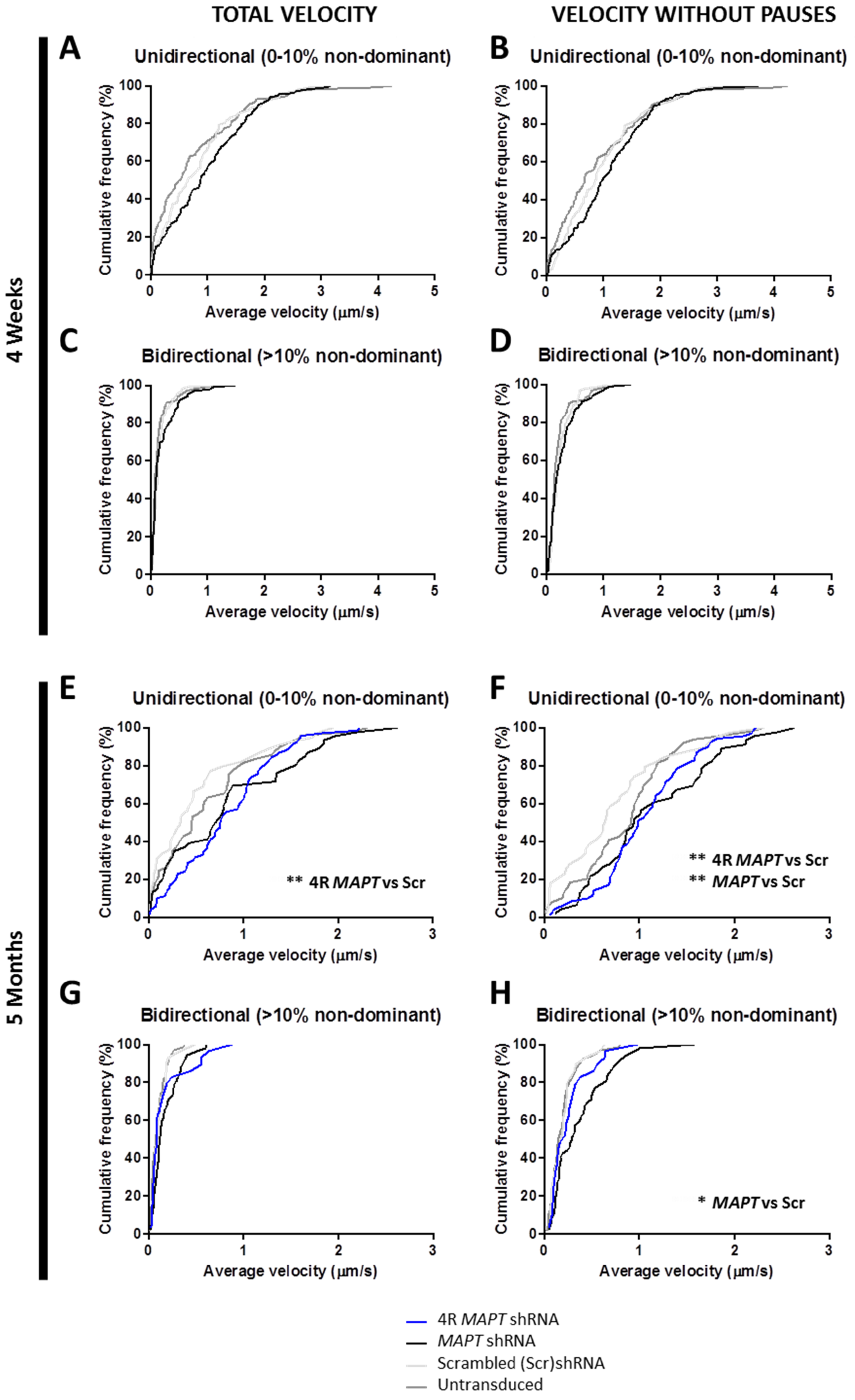
TOTAL VELOCITY
VELOCITY WITHOUT PAUSES
4 Weeks
5 Months
A
Unidirectional (0-10% non-dominant)
B
Unidirectional (0-10% non-dominant)
C
Bidirectional (>10% non-dominant)
D
Bidirectional (>10% non-dominant)
E
Unidirectional (0-10% non-dominant)
** 4R MAPT vs Scr
F
Unidirectional (0-10% non-dominant)
** 4R MAPT vs Scr
** MAPT vs Scr
G
Bidirectional (>10% non-dominant)
H
Bidirectional (>10% non-dominant)
* MAPT vs Scr
Cumulative frequency (%)
Average velocity (μm/s)
4R MAPT shRNA
MAPT shRNA
Scrambled (Scr)shRNA
Untransduced

Figure S6. Stratified mitochondrial axonal transport data for iPSC-derived dopaminergic neuronal cultures, related to Figure 6. Legend for all parts at bottom of figure. Cumulative frequency (%) graphs of average mitochondrial velocity in dopaminergic neuronal cultures after stratification by directionality.

(A-D) Stratified axonal transport data for cultures around four weeks post-transduction. (A) Total velocity measurements for unidirectional mitochondria; (B) velocity without pause periods for unidirectional mitochondria; (C) Total velocity measurements for bidirectional mitochondria; (D) velocity without pause periods for bidirectional mitochondria. Median velocities (IQR): (A) total MAPT shRNA, 0.882 (1.187) µm/s; non-targeting shRNA, 0.658 (0.908) µm/s; untransduced, 0.530 (1.139) µm/s; (B) total MAPT shRNA, 0.992 (1.011) µm/s; non-targeting shRNA, 0.869 (0.965) µm/s; untransduced, 0.671 (1.134) µm/s; (C) total MAPT shRNA, 0.096 (0.182) µm/s; non-targeting shRNA, 0.121 (0.130) µm/s; untransduced, 0.080 (0.092) µm/s; (D) total MAPT shRNA, 0.173 (0.256) µm/s; non-targeting shRNA, 0.195 (0.202) µm/s; untransduced, 0.146 (0.141) µm/s. Kruskall-Wallis test found a significant statistical difference between medians for (A) ($p=0.0097$, KW statistic=9.264) but Dunn's multiple comparisons post test showed no statistical difference from non-targeting shRNA control (total MAPT shRNA, adjusted $p=0.2327$). Kruskall-Wallis tests for (B-D) found no significant difference between medians: (B) $p=0.0502$, KW statistic=5.984; (C) $p=0.1458$, KW statistic=3.851; (D) $p=0.1786$, KW statistic=3.445. Number of mitochondria per condition (unidirectional/bidirectional): total MAPT shRNA, 133/102; non-targeting shRNA, 102/93; untransduced, 112/100.

(E-H) Stratified axonal transport data for cultures around five months post-transduction. (E) Total velocity measurements for unidirectional mitochondria; (F) velocity without pause periods for unidirectional mitochondria; (G) Total velocity measurements for bidirectional mitochondria; (H) velocity without pause periods for bidirectional mitochondria. Median velocities (IQR): (E) 4R *MAPT* shRNA, 0.760 (0.752) µm/s; total *MAPT* shRNA, 0.723 (1.202) µm/s; non-targeting 0.344 (0.573) µm/s; untransduced, 0.458 (0.718) µm/s; (F) 4R *MAPT* 0.990 (0.626) µm/s; total *MAPT* shRNA, 0.946 (1.056) µm/s; non-targeting 0.641 (0.827) µm/s; untransduced, 0.913 (0.660) µm/s; (G) 4R *MAPT* 0.085 (0.131) µm/s; total *MAPT* shRNA, 0.120 (0.193) µm/s; non-targeting 0.093 (0.113) µm/s; untransduced, 0.076 (0.106) µm/s; (H) 4R *MAPT* 0.214 (0.215) µm/s; total *MAPT* shRNA, 0.294 (0.389) µm/s; non-targeting 0.184 (0.154) µm/s; untransduced, 0.153 (0.145) µm/s. Kruskall-Wallis tests found significant statistical differences between medians for all graphs: (E) $p=0.0075$, KW statistic=11.97; (F) $p=0.0030$, KW statistic=13.96; (G) $p=0.0113$, KW statistic=11.09; (H) $p=0.0022$, KW statistic=14.59. Asterisks represent significant statistical difference from non-targeting shRNA control in Dunn's multiple comparisons tests: (E) 4R *MAPT* shRNA, $p=0.0048$; (F) 4R *MAPT* shRNA, $p=0.0033$; total *MAPT* shRNA, $p=0.0078$; (G) no significance in post test; (H) total *MAPT* shRNA, $p=0.0327$. Number of mitochondria per condition (unidirectional/bidirectional): 4R *MAPT* shRNA, 70/29; total *MAPT*, 52/46; non-targeting shRNA, 39/29; untransduced, 49/66.

.

Table S1: iPSC clones used in this study, Related to Figure 1.

OPDC (StemBANCC) codes	Age at biopsy	Sex	*MAPT* genotype	Clones used	Paper ID	Reprogramming Method	Previous Characterisation
NHDF	44	F	H1/H2	NHDF-1<br><br>NHDF-2	1A<br><br>1B	Retrovirus<br><br>Retrovirus	Hartfield et al. (2014)<br><br>Hartfield et al. (2014)
AH016	80	M	H1/H2	AH016-3<br>AH016-6<br>AH016-10	2A<br>2B<br>2C	Retrovirus<br>Retrovirus<br>Retrovirus	Sandor et al. (2017)<br>-<br>-
OX1 (SFC841)	36	M	H1/H2	OX1-19<br><br>SFC841-03-01<br>SFC841-03-02	3A<br><br>3B<br>3C	Retrovirus<br><br>CytoTune<br>CytoTune	van Wilgenburg et al. (2013)<br>(Dafinca et al., 2016)<br>-

Supplemental Experimental Procedures

Genotyping, sequencing and sub-cloning

Genomic DNA was extracted from cultured cells using the Illustra tissue and cells genomicPrep Mini Spin Kit (GE Healthcare, Amersham, UK). *MAPT* H1/H2 genotyping reactions were performed using AmpliTaq Gold DNA Polymerase with Gold Buffer and 2.5 mM $MgCl_2$ (Applied Biosystems), with primers to amplify around the 238 bp indel in *MAPT* intron 9 (Table S2). To determine the sequence around *MAPT* exons 9 and 10, initial amplification PCRs from genomic DNA using AmpliTaq Gold were cleaned up using FastAP (ThermoScientific) and Exonuclease I (New England BioLabs (NEB)) before a second single-primer reaction with BigDye Terminator v3.1 kit (Applied Biosystems) and sequencing on a 3730xl DNA Analyzer (Applied Biosystems) at the Zoology Sequencing Facility, University of Oxford.

Products from allele-specific amplification of the region around *MAPT* exon 10 by AmpliTaq Gold were ligated into pGEM-T Easy (Promega) and electroporated into NEB 10-beta electrocompetent *E. coli* (NEB) prior to blue-white selection on ampicillin-agar. Plasmids were isolated using the QIAprep Spin Miniprep kit (QIAGEN) and sequenced directly with BigDye.

Induced pluripotent stem cells

iPSC lines used in this manuscript are detailed in Table S1. The existing lines NHDF-1 and NHDF-2 (Hartfield et al., 2014) were used as clones 1A and 1B in this study. Further lines were generated from two MAPT H1/H2 healthy control individuals screened within the Oxford Parkinson's Disease Cohort following reprogramming either by retroviral delivery as previously described (clones 2A, 2B, 2C, 3A)(Hartfield et al., 2014; van Wilgenburg et al., 2014) or using the CytoTune-iPS Sendai Reprogramming kit (Invitrogen) (Fernandes et al., 2016) (clones 3B, 3C). Clones were adapted to feeder-free culture conditions in mTeSR™1 (StemCell Technologies), on hESC-qualified Matrigel-coated plates (BD), and routinely passaging as clumps using 0.5 mM EDTA in PBS (Beers et al., 2012). Large-scale SNP-QCed batches were frozen at p15-25 and used for experiments within a minimal number of passages post-thaw to ensure consistency.

QC analyses for previously unpublished clones are shown in Figure S1-S2, and were carried out as described previously (Hartfield et al., 2014) (Fernandes et al., 2016). FACs for pluripotency markers TRA-1-60 and Nanog (B119983, IgM-488, Biolegend; 2985S, IgG-647, Cell Signaling, with appropriate isotype controls, using the same concentration and supplier), was measured using a FACS Calibur (Becton Dickinson), and analysis using FlowJo.

Silencing of retrovirally-delivered reprogramming genes was assessed by quantitative RT-PCR as previously described (Hartfield et al., 2014). Clearance of Cytotune Sendai virus-delivered reprogramming genes was performed as previously described (Fernandes et al., 2016) by RT-PCR, run on a 1.5% agarose gel with Log2 ladder (NEB). Positive controls (fibroblasts infected 5 days previously) were always run in parallel. Primers were SeV F: GGATCACTAGGTGATATCGAGC, R: ACCAGACAAGAGTTTAAGAGATATGTATC 181bp; SOX2 F: ATGCACCGCTACGACGTGAGCGC, R: AATGTATCGAAGGTGCTCAA 451bp; KLF4 F: TTCCTGCATGCCAGAGGAGCCC, R: AATGTATCGAAGGTGCTCAA 410bp; c-MYC F:

TAACTGACTAGCAGGCTTGTCG, R: TCCACATACAGTCCTGGATGATGATG 532bp; OCT4 F: CCCGAAAGAGAAAGCGAACCAG, R: AATGTATCGAAGGTGCTCAA 483bp; β-Actin control Eurogentec 92 bp.

Assessment of conformity to pluripotent gene expression profile was performed using Illumina's Human-HT-12-v4 expression BeadChip and Pluritest (Muller et al., 2011) (pluritest.org).

Genome integrity and cell-line tracking was assessed by Illumina Human CytoSNP-12v2.1 beadchip array (~300,000 markers) or OmniExpress24 array (700,000 markers), with genomic DNA made using an All-Prep kit (Qiagen) and analysis used GenomeStudio and Karyostudio software (Illumina). The accession number for the Illumina SNP genotype and HT12v4 expression array datasets reported in this paper is GEO: GSE99125.

Differentiation of induced pluripotent stem cells to dopaminergic neuronal cultures

All cell cultures were maintained at 37ºC, 5% CO_2. Induced pluripotent stem (iPS) cells were maintained in 6-well plates coated with hESC-qualified Matrigel (Corning) with mTeSR1 medium (StemCell Technologies) changed daily. ROCK inhibitor (10 µM Y-27632) (Tocris Bioscience) was used for 24 hours after single cell passaging performed with TrypLE Express incubation (Life Technologies). iPS cells were differentiated into dopaminergic neuronal cultures according to a modified protocol of Kriks et al (Kriks et al., 2011). Prior to commencing differentiation, iPS cells were passaged as single cells and seeded in 6-well plates coated with Geltrex (Life Technologies) then grown to confluency. Basal media are as follows (Life Technologies unless stated): KO DMEM KSR = Knockout DMEM, Knockout serum replacement, 1X non-essential amino acids, 2 mM L-glutamine, 10 µM 2-mercaptoethanol (Sigma); NNB = Neurobasal medium, 0.5X N2 supplement, 0.5X B27 supplement, 2mM L-glutamine; NB = Neurobasal medium, 1X B27 supplement, 2mM L-glutamine. Differentiation factors added to basal media are as follows: 100 nM LDN-193189 (Sigma), 10 µM SB-431542 (Tocris Bioscience), 100 ng/ml recombinant sonic hedgehog C24II (R&D Systems), 2 µM purmorphamine (Calbiochem), 100 ng/ml fibroblast growth factor 8a (R&D Systems), 3 µM CHIR-99021 (Tocris Bioscience), 20 ng/ml brain-derived neurotrophic factor (Peprotech), 20 ng/ml glial cell line-derived neurotrophic factor (Peprotech), 1 ng/ml transforming growth factor β3 (Peprotech), 10 µM DAPT (abcam), 200 µM ascorbic acid (Sigma), 500 µM dibutyryl cAMP (Sigma). Medium containing differentiation and neurotrophic factors was fully changed every two days with half change every other day until day 20 of the protocol when cells were dissociated with StemPro Accutase (Life Technologies) and re-plated onto poly-L-ornithine with laminin and fibronectin or Geltrex in the desired format and density per experiment, ranging from spots of $5x10^4$ cells to an even monolayer of $3x10^5$ cells/cm^2. Cultures were treated with 1 µg/ml mitomycin C in NB medium for 1 hour to remove proliferating cells and washed with neurobasal medium before returning to fresh maturation medium. After a full medium change three days later to remove dead cells, medium was half changed every 2-3 days for the remaining period of maturation up to DIV190. For biochemical analysis of protein or RNA, cultures were washed in PBS, detached by scraping in fresh PBS, micro-centrifuged (1200 x *g*, 5 min, 4ºC), snap frozen on dry ice and stored at -80ºC until ready for lysis.

Primers used for PCR, sequencing and cloning

Primer Name	Primer Sequence (5'-3')	Primer Purpose
tau_indel_F	GGAAGACGTTCTCACTGATCTG	Genotyping H1/H2 (238 bp indel)
tau_indel_R	AAGAGTCTGGCTTCAGTCTCTC	
M13_F	GTAAAACGACGGCCAGT	Sequencing of sub-clones in pGEM-T Easy
M13_R	CAGGAAACAGCTATGAC	
MAPT_Int9seqF	TGTGAAGTGAGGACCTGCAA	Sequencing upstream of *MAPT* exon 10
MAPT_Int9seqR	AAAAGGATGAGTGACACGCC	
MAPTEx10seqF	CTCTGCCAAGTCCGAAAGTG	Sequencing *MAPT* exon 10 and environs
MAPTEx10seqR	GGTCCGTCATCTGCCCTATT	
MAPT_int9_H1F	GAAATGCAGTCGTGGGAGAC	Generation of H1 sub-clone (with MAPTEx10seqR)
MAPT_int9_H2F	TGGTTTTCTATTTCACAGCCCC	Generation of H2 sub-clone (with MAPTEx10seqR)
RBM4_F	GCCGCCATTTTAGCGTTTTG	SyBr Green qPCR of *RBM4*
RBM4_R	CACATTCCAGCACCTTCCCA	
PTBP1_F	TTGGGTCGGTTCCTGCTATT	SyBr Green qPCR of *PTBP1*
PTBP1_R	CGTCAGATCCCCGCTTTGT	

Allele-specific qRT-PCR

Specificity for the reactions was achieved with 5X SNP1 probe concentration, 3X SNP9ii probe concentration and 900 nM primers. H1:H2 ratios were calculated as follows: 2^(-1.194*[(cDNA ΔC_T H1-H2) – (Mean genomic ΔC_T H1-H2)] for SNP1 and 2^(-1.043*[(cDNA ΔC_T H1-H2) – (Mean genomic ΔC_T H1-H2)] for SNP9ii. For midbrain samples, the mean of the genomic ΔCT H1-H2 values for the eight iPS clones was used. Specific H1:H2 ratios for inclusion of exon 3 and exon 10 in MAPT transcripts were determined by dividing by the respective H1:H2 ratio for total MAPT transcripts.

TaqMan Gene Expression Assays

Gene/Assay Description	Assay ID
MAPT exon3+ transcripts (exon3-4)	Hs00902315_m1 FAM/MGB/NFQ
MAPT exon 10+ transcripts (exon9-10)	Hs00902312_m1 FAM/MGB/NFQ
MAPT all transcripts (exon12-13)	Hs00902194_m1 FAM/MGB/NFQ
GAPDH	Hs02758991_g1 VIC/MGB/NFQ
HPRT1	Hs02800695_m1 VIC/MGB/NFQ
ACTB	Hs01060665_g1 VIC/MGB/NFQ

Primers and probes for allele-specific qRT-PCR TaqMan expression assays.

Primer/Probe Name	Primer/Probe Sequence (5'-3')	Primer/Probe Purpose
MAPT exon 0 F	CCTCGCCTCTGTCGACTATC	MAPT all transcripts allele-specific (exon 0-1)
MAPT exon 1 R	TACGTCCCAGCGTGATCTTC	
MAPT exon 0 F	CCTCGCCTCTGTCGACTATC	MAPT exon 3+ transcripts allele-specific (exon 0-3)
MAPT exon 3 R	GTCACATCTTCCGCTGTTGG	
MAPT intron 0 F	CCCCAACACTCCTCAGAACT	MAPT allele-specific exon 1 genomic
MAPT exon 1 R	TACGTCCCAGCGTGATCTTC	
MAPT exon 9 F	AAGAGCCGCCTGCAGACA	MAPT exon 10+ transcripts allele-specific (exon 9-10)
MAPT exon 10 R	GGACGTTGCTAAGATCCAGCTT	
MAPT exon 9 F	AAGAGCCGCCTGCAGACA	MAPT allele-specific exon 9 genomic
MAPT intron 9 R	ACCTCCATGCACAGTCCCA	
SNP1 H1 Probe (FAM/MGB/NFQ)	CTGGTTCAAAGTTC	
SNP1 H2 Probe (VIC/MGB/NFQ)	TGGTTCAAAGCTCAC	
SNP9ii H1 Probe (FAM/MGB/NFQ)	TTGGACTTGACATTCT	
SNP9ii H2 Probe (VIC/MGB/NFQ)	CTTGGACTTGACGTTCT	

Western blotting

Cell pellets were sonicated in RIPA buffer (50 mM tris-HCl, pH 7.4, 150 mM NaCl, 1% (v/v) Triton X-100, 1% (w/v) sodium deoxycholate, 0.1% (w/v) SDS) for standard Western blotting, or in TBS (20 mM tris-HCl, pH 7.4, 140 mM NaCl) when protein dephosphorylation would subsequently be performed; cOmplete mini protease inhibitors (Roche) were added to both buffers. After 30 minutes incubation on ice, the soluble fraction was isolated by micro-centrifugation (1200 x *g*, 20 min, 4 ºC). Protein concentrations were determined by BCA assay (ThermoScientific) with a BSA calibration curve.

For protein dephosphorylation to reveal tau isoforms, 15-20 µg protein samples were incubated with lambda phosphatase (NEB) (20 units/µl, 60 min, 30ºC) followed by denaturation in Laemmli sample buffer (Laemmli, 1970) (95 ºC, 10 min). Non-dephosphorylated blots had 5 µg protein loading. Protein separation was achieved using 10% (dephosphorylated protein blots) or 4-15% (other blots) Criterion TGX polyacrylamide gels (Bio-Rad) in a tris-glycine running buffer, with transfer to a PVDF membrane using the TransBlot-Turbo Transfer System (Bio-Rad).

Blots were blocked with 5% milk (Sigma) in TBS with 0.1% (v/v) Tween 20 (TBST), incubated with primary antibody in 1% milk-TBST (overnight, 4ºC), washed three times with TBST, probed with horseradish peroxidase (HRP)-conjugated secondary antibody in 1% milk-TBST for 1 hour at room temperature, and washed four times in TBST. Immobilon Western Chemiluminescent HRP Substrate (Millipore) was added and chemiluminescence detected using the ChemiDoc Touch System (Bio-Rad). Bound proteins were removed using Restore Western Blot Stripping Buffer (ThermoScientific, 15 min) and immunodetection was repeated using additional antibodies. Primary antibodies used in this study: β3-tubulin (TUJ1) (1:1000, Covance, MMS-435P), tyrosine hydroxylase (1:2000, Millipore, ab152), HRP-conjugated β-actin (1:20,000, abcam, ab49900),

tau-1 clone PC1C6 (1:1000, Millipore, MAB3420), 4R tau repeat isoform RD4 (1:250, Millipore, 05-804), 2N tau clone 71C11 (1:1000, Covance, Sig-39408), tau-5 (1:5000, NeoMarkers, MS-247-P). Secondary antibodies used in this study: HRP-conjugated goat anti-mouse (1:5000, Bio-Rad, 170-6516), HRP-conjugated goat anti-rabbit (1:5000, Bio-Rad, 170-6515).

Immunocytochemistry

Cultures grown on glass coverslips were washed with PBS, fixed with 4% (w/v) paraformaldehyde in PBS for 10-15 min and washed three times with PBS. Cell permeabilisation and protein blocking were performed with 10% goat serum in PBS 0.1% Triton X-100 (PBST, 2 hours) prior to incubation with primary antibodies in PBST with 1% goat serum (4 ºC overnight). Coverslips were washed three times with PBST and incubated with Alexa Fluor-labelled secondary antibodies for 1 hour to enable visualisation. Coverslips were washed with PBS and incubated with 1 µg/ml DAPI (Sigma) in PBS for 5 min then washed again with PBS. Coverslips were mounted on glass slides with FluorSave (Calbiochem) and cells imaged using an EVOS FL Auto Imaging System (Life Technologies). Antibodies used in this study: TUJ1 (1:500, Covance, MMS-435P), tyrosine hydroxylase (1:250, Millipore, ab152), Alexa-488 goat anti-mouse (1:500, Invitrogen, A11001), Alexa-594 goat anti-rabbit (1:500, Invitrogen, A11012).

RNA extraction and cDNA synthesis

Midbrain samples were from healthy control subjects without any neurological symptoms obtained from the UK MRC Control collection run by Oxford Brain Bank, University of Oxford. Samples were assessed by a neuropathologist and any found to have protein deposition or morphological abnormalities except minor, age-related Alzheimer's changes were excluded from the analysis. Full ethical approval (REC15/SC/0639) and written informed consent to brain and spinal cord donation were obtained from the relatives of all potential tissue donors dying in the Oxford University Hospital Foundation Trust Hospitals.

Samples of frozen post-mortem human midbrain were obtained for *MAPT* H1/H2 individuals. Samples were composed of several cryostat-generated horizontal sections that all included the substantia nigra. Before RNA extraction, samples were homogenised in RNeasy Buffer RLT with 1% 2-mercaptoethanol using a TissueRuptor (QIAGEN).

RNA was extracted from culture cell pellets or midbrain homogenate using RNeasy Micro/Mini kits (QIAGEN) according to manufacturer's instructions. RNA concentration was determined by Nanodrop (ThermoScientific). RNA integrity number was determined for midbrain samples using a Eukaryote Total RNA Pico Assay on a 2100 Bioanalyzer System (Agilent Technologies) (n=9; mean = 6.6; median = 6.8, IQR = 1.6). First-strand cDNA synthesis was performed using SuperScript VILO MasterMix (Invitrogen) according to manufacturer's instructions with a two-hour synthesis step.

Quantitative real-time polymerase chain reaction (qRT-PCR)

All qRT-PCR assays were run as 20 µl reactions on a StepOnePlus System using either TaqMan Gene Expression Master Mix or Fast SYBR Green Master Mix (Applied Biosystems) with 200 nM of each primer. 5 ng or 10 ng cDNA was included in each reaction. TaqMan Gene Expression Assays were used to determine

expression of specific *MAPT* transcripts and multiplexed with VIC-labelled assays for housekeeping genes (Applied Biosystems, Table S3). A common threshold value of 0.1 was set for all assays (except allele-specific assays) to enable comparison. Expression of total *MAPT*, *RBM4* and *PTBP1* were determined as 2^–(Assay C_T – housekeeper geometric mean). *MAPT* isoform-specific assays were normalised to Total *MAPT* instead of housekeeper assays, with exon % inclusion determined by the formula [2^–(*MAPT* isoform assay C_T – Total *MAPT* C_T)]*100.

RNA electrophoretic mobility shift assay

SK-N-F1 cells were grown in 15 cm dishes for 48 hours and harvested by gentle scraping. The cytoplasmic fraction was first extracted using cold lysis buffer: 10 mM HEPES (Sigma), 10 mM KCl (Ambion), 0.1 mM EDTA (Ambion), 0.1 mM EGTA (Sigma), Halt protease and phosphatase inhibitor cocktails (Thermo Scientific) and 0.67 % IGEPAL CA-630 (Sigma). The nuclear pellet was washed and lysed using cold nuclear lysis buffer (20 mM HEPES, 400 mM KCl, 1 mM EDTA, 1mM EGTA and Halt protease and phosphatase inhibitor cocktails.

RNA electrophoretic mobility shift assay (EMSA) was carried out using RNA oligonucleotides biotinylated at the 3' end, SK-N-F1 nuclear lysates and the LightShift Chemiluminescent RNA EMSA Kit (Pierce) according the manufacturer's protocol. RNA oligonucleotides containing intron 10 WT sequence: H1 (5' TGCATAGAATAAATCCTT**CTT**GGGCTCTCAGGA 3') and intron 10 ΔCTT sequence (5' TGC ATA GAA TAA ATC CTT GGG CTC TCA GGA 3'). RNA probes contain the same sequence with a 15 atom tetraethylene glycol linker biotin at the 3' end of the oligonucleotide (Integrated DNA Technologies).

RNA-Protein pull-down

RNA pull-down of RNA binding proteins was performed using the Magnetic RNA Protein Pull-Down kit (Pierce) according to the manufacturer's instructions. 50pmol of biotinylated RNA oligonucleotides were allowed to bind streptavidin magnetic beads and incubated with 40µg of SKNF1 nuclear enriched lysate for 1 hour at 4ºC. Unbound proteins were washed off and RNA-protein complexes were dissociated from the magnetic beads by boiling in 5X Laemmli buffer for 10 min. The isolated RNA-protein complexes were loaded onto a 10% Tris-glycine SDS-PAGE for western blotting. Blots were probed with anti-PTBP1 (1:1000, Abcam, ab30317) and anti-RBM4 (1:500, Abcam, ab130624) antibodies

Generation of lentiviral constructs and production of lentiviral particles

shRNA sequences were identified to target *MAPT* exon 10 (corresponding to 4R tau), *MAPT* exons 12-13 (constitutive exons for targeting total *MAPT*) and no known RefSeq transcript (non-targeting shRNA). Incorporation of shRNA sequences into lentiviral plasmids was performed using a modification of the PCR-based protocol of Harper and Davidson (2005), using Gibson Assembly to perform all construction steps in one (Fig. S3). The following DNA molecules were joined using Gibson Assembly Master Mix (NEB): pRRL.sin.wpre fragment of *Spe*I-HF/*Sal*I-HF double digest (NEB) of pRRL.sin.U6.shRNA.cPPT.CMV.EGFP.wpre (kind gift from Dr Óscar Cordero Llana); PCR of U6 promoter from the same construct, adding a specific shRNA sequence with the reverse primer; PCR of RRE.cPPT.pEF1α

from CSii-EF-MCS (a gift from Dr H. Miyoshi, RIKEN BioResource Center DNA Bank); PCR of EBFP2 from pBAD-EBFP2 (a gift from Robert Campbell, Addgene #14891)(Ai et al., 2007). All PCRs were performed using KAPA HiFi HotStart ReadyMix (KAPA Biosystems); see Table S5 for primer sequences. All DNA molecules were separated by agarose gel electrophoresis (2% for shRNA PCRs and 1% for all others), purified by QIAquickGel Extraction Kit (QIAGEN) and quantified by Nanodrop before Gibson Assembly. Assembly products were used to transform One Shot TOP10 Chemically Competent *E. coli* (Invitrogen) for growth and selection, then One Shot Stbl3 Chemically Competent *E. coli* (Invitrogen) following verification and sequencing.

For third generation production of lentiviral particles, the following plasmids were prepared by PureLink HiPure Plasmid Maxiprep kit (Invitrogen) and transfected into HEK293T cells in 15 cm dishes: 10 µg shRNA/EBFP2 lentiviral vector; 10 µg pMDLg/pRRE (a gift from Didier Trono, Addgene #12251)(Dull et al., 1998); 2 µg pRSV-Rev (a gift from Didier Trono, Addgene #12253); 3.4 µg pMD2.g (a gift from Didier Trono, Addgene #12259) encoding vesicular stomatitis virus glycoprotein (VSV-G) for lentiviral pseudotyping.

HEK293T cells were transfected by calcium phosphate transfection, whereby DNA was incubated with 125 mM $CaCl_2$, 25 mM HEPES, 140 mM NaCl, 0.75 mM Na_2HPO_4 (Sigma) for 30 min before adding to HEK293T cultures. On day two the culture medium was replaced, containing 10 mM sodium butyrate (Sigma), then collected eight hours later as the first harvest. Following replacement, a second harvest of medium was performed at the end of day three and pooled with the first harvest. Viral medium was centrifuged to remove debris (1,000 x *g*, 5 min), 0.45 µm filtered, then centrifuged overnight to pellet viral particles (6,000 x *g*, 4ºC). On day four the pellet was re-suspended in ice-cold PBS, ultra-centrifuged (20,000 x *g*, 90 min, 4ºC) and finally re-suspended in 20 mM Tris base, 100 mM NaCl, 10 g/l sucrose, 10 g/l D-mannitol (Sigma); brief centrifugation (1000 x *g*, 5 min, 4ºC) removed persistent debris before aliquoting for -80ºC storage.

Viral titres were determined by transduction of HEK293T cells with serial dilutions, followed by flow cytometry determination of the percentage of EBFP2-positive cells 72 hours post-transduction.

Primers for construction of lentiviral shRNA plasmids by Gibson Assembly.

Primer Name	Primer Sequence (5’-3’) with shRNA sequence shown in bold	Primer Position
LenGib_1F	TTCGCCCTTACGCTCTAGAAC	Before U6
LenGib_x10R	TGTCCCTCCTCGAGAAAAAA**GGTGCAGATAATTAATAAGTTCGCTTATTAATTATCTGCACCTTC**GCAAACAAGGCTTTTCTCCAAG	To add shRNA sequence against exon 10+ *MAPT*
LenGib_TotR	TGTCCCTCCTCGAGAAAAAA**CCAGGTGGAAGTAAAATCTGATTCGTCAGATTTTACTTCCACCTGGC**GCAAACAAGGCTTTTCTCCAAG	To add shRNA sequence against total *MAPT*
LenGib_Scr	TGTCCCTCCTCGAGAAAAAA**GAAGGCTCGTCGCACTAATTTCGATTAGTGCGACGAGCCTTCTTC**GCAAACAAGGCTTTTCTCCAAG	To add scrambled shRNA sequence
LenGib_2F	TTTTTTCTCGAGGAGGGACAATTGGAGAAGTG	Before RRE
LenGib_2R	CTGCAGAATTCTCGAGACCG	After pEF1α
LenGib_3F	CGGTCTCGAGAATTCTGCAGGCCACCATGGTGAGCAAGGGCGAGG	Start of EBFP2
LenGib_3R	TGTAATCCAGAGGTTGATTGTCGACTTACTTGTACAGCTCGTCCATG	End of EBFP2

Mitochondrial axonal transport imaging

Cultures of iPS-derived dopaminergic neuronal cultures were transduced with lentiviral particles encoding shRNAs on DIV20 following re-plating onto Geltrex-coated coverslips. Cultures were imaged on a Nikon Eclipse TE-2000-U fluorescent microscope with heated chamber at 37ºC and delivery of 5% CO_2/Air for up to one hour with a 60X immersion objective (Nikon Plan APO VC, 60x/1.40 oil, 0.17, DIC, N2).

Image analysis

Kymograph time-space plots were generated from blinded videos in Fiji software (National Institutes of Health) using the Multiple Kymograph plugin and the tsp050706 macro according to its protocol (http://www.embl.de/eamnet/html/body_kymograph.html). For transduced coverslips, only EBFP2-positive axons were chosen for analysis. Within the multiple kymograph, a segmented line was drawn along the path of each mitochondrion and velocities were determined using the 'read velocities from tsp' macro. Due to the complex network that develops in extended culture, the position of the cell body was not identified so directionality could not be determined. Mitochondria were classed as motile if they moved more than 2 µm during the imaging period, corresponding to the approximate length of a mitochondrion (Vossel et al., 2015). Pauses were determined as individual parts of the path where a mitochondrion moved 0-1 pixels and/or had a velocity <0.12 pixels/s (<0.0139 µm/s) corresponding to the minimum velocity needed to reach the overall threshold of motility in the 150 s imaging window. Data are presented as average velocity (µm/s) for the measured period and also the same average velocity calculation but with pause periods removed.

Supplemental Information References

Ai, H.W., Shaner, N.C., Cheng, Z., Tsien, R.Y., and Campbell, R.E. (2007). Exploration of new chromophore structures leads to the identification of improved blue fluorescent proteins. Biochemistry *46*, 5904-5910.

Beers, J., Gulbranson, D.R., George, N., Siniscalchi, L.I., Jones, J., Thomson, J.A., and Chen, G. (2012). Passaging and colony expansion of human pluripotent stem cells by enzyme-free dissociation in chemically defined culture conditions. Nature protocols *7*, 2029-2040.

Dafinca, R., Scaber, J., Ababneh, N.a., Lalic, T., Weir, G., Christian, H., Vowles, J., Douglas, A., Fletcher-Jones, A., Browne, C., *et al.* (2016). C9orf72 Hexanucleotide Expansions are Associated with Altered ER Calcium Homeostasis and Stress Granule Formation in iPSC-Derived Neurons from Patients with Amyotrophic Lateral Sclerosis and Frontotemporal Dementia. Stem cells (Dayton, Ohio).

Dull, T., Zufferey, R., Kelly, M., Mandel, R.J., Nguyen, M., Trono, D., and Naldini, L. (1998). A third-generation lentivirus vector with a conditional packaging system. J Virol *72*, 8463-8471.

Fernandes, H.J., Hartfield, E.M., Christian, H.C., Emmanoulidou, E., Zheng, Y., Booth, H., Bogetofte, H., Lang, C., Ryan, B.J., Sardi, S.P., *et al.* (2016). ER Stress and Autophagic Perturbations Lead to Elevated Extracellular alpha-Synuclein in GBA-N370S Parkinson's iPSC-Derived Dopamine Neurons. Stem cell reports *6*, 342-356.
Harper, S.Q., and Davidson, B.L. (2005). Plasmid-based RNA interference: construction of small-hairpin RNA expression vectors. Methods Mol Biol *309*, 219-235.
Hartfield, E.M., Yamasaki-Mann, M., Ribeiro Fernandes, H.J., Vowles, J., James, W.S., Cowley, S.A., and Wade-Martins, R. (2014). Physiological characterisation of human iPS-derived dopaminergic neurons. PLoS One *9*, e87388.
Kriks, S., Shim, J.W., Piao, J., Ganat, Y.M., Wakeman, D.R., Xie, Z., Carrillo-Reid, L., Auyeung, G., Antonacci, C., Buch, A., *et al.* (2011). Dopamine neurons derived from human ES cells efficiently engraft in animal models of Parkinson's disease. Nature *480*, 547-551.
Laemmli, U.K. (1970). Cleavage of structural proteins during the assembly of the head of bacteriophage T4. Nature *227*, 680-685.
Muller, F.J., Schuldt, B.M., Williams, R., Mason, D., Altun, G., Papapetrou, E.P., Danner, S., Goldmann, J.E., Herbst, A., Schmidt, N.O., *et al.* (2011). A bioinformatic assay for pluripotency in human cells. Nature methods *8*, 315-317.
van Wilgenburg, B., Moore, M.D., James, W.S., and Cowley, S.A. (2014). The productive entry pathway of HIV-1 in macrophages is dependent on endocytosis through lipid rafts containing CD4. PLoS One *9*, e86071.
Vossel, K.A., Xu, J.C., Fomenko, V., Miyamoto, T., Suberbielle, E., Knox, J.A., Ho, K., Kim, D.H., Yu, G.Q., and Mucke, L. (2015). Tau reduction prevents Abeta-induced axonal transport deficits by blocking activation of GSK3beta. J Cell Biol *209*, 419-433.
